# Proteoglycan-4 potentiates the antitumor efficacy of regorafenib in an orthotopic model of hepatocellular carcinoma

**DOI:** 10.1186/s13046-025-03575-5

**Published:** 2025-12-01

**Authors:** Livianna Carrieri, Anas Munir, Giusi Caragnano, Davide Guido, Grazia Serino, Emanuele Piccinno, Domenico Mastronardi, Giusy Bianco, Nicolò Schena, Raffaele Armentano, Francesco Dituri, Gianluigi Giannelli

**Affiliations:** National Institute of Gastroenterology, IRCCS “S. de Bellis” Research Hospital, Via Turi 27, Castellana Grotte, BA, 70013 Italy

**Keywords:** Hepatocellular carcinoma, Proteoglycans, Regorafenib, Experimental liver cancer model

## Abstract

**Background:**

Hepatocellular carcinoma (HCC) presents a significant therapeutic challenge, as current treatment options provide limited long-term benefits due to issues surrounding their effectiveness and associated adverse effects. Our previous research demonstrated that Proteoglycan-4 (PRG4) enhances the anti-proliferative effect of the multi-kinase inhibitor regorafenib in simple in vitro two-dimensional HCC models. In this study, we aimed to investigate the potential adjuvant role of PRG4 in improving the efficacy of regorafenib within both three-dimensional in vitro and in vivo HCC models.

**Methods:**

Human HCC cells were engineered to stably overexpress PRG4. The effects of PRG4 on cell proliferation, both alone and in combination with regorafenib, were tested in monolayer cultures, Matrigel-embedded spheroids, and an orthotopic xenograft HCC mouse model. Additionally, transcriptomic profiling of spheroids generated from control or PRG4-overexpressing HCC cells, either untreated or treated with regorafenib, was performed.

**Results:**

PRG4 expression partially inhibited HCC tumor growth in vivo and enhanced regorafenib antiproliferative activity, leading to a near-complete tumor regression. This synergistic PRG4 + regorafenib interaction in impairing HCC cell growth was further confirmed in 2D and 3D HCC models in vitro. In addition, PRG4 restrained angiogenesis by hindering endothelial tubulogenesis in vitro. By transcriptomic analysis of matrigel-embedded HCC cell spheroids exposed to PRG4 and/or regorafenib, PDGF pathway emerged as a target of PRG4 + regorafenib, corroborating the role of PRG4 in impairing angiogenesis. The G_0_/G_1_ phase of the cell cycle was more delayed in spheroids exposed to both PRG4 and regorafenib compared to those treated with regorafenib alone, relative to untreated cells.

**Conclusions:**

PRG4 demonstrated antitumor activities in vivo and shows promise as an adjuvant to enhance therapeutic interventions in HCC.

**Supplementary Information:**

The online version contains supplementary material available at 10.1186/s13046-025-03575-5.

## Introduction

Hepatocellular carcinoma (HCC) accounts for nearly three-quarters of primary liver cancer cases worldwide. It is associated with a generally poor prognosis and exhibits a rising incidence, particularly in Western Europe and North America. The incidence and mortality rates of this neoplasm are roughly equivalent to 9.3 and 8.5 per 100,000 person years, respectively [1]. While the hepatitis B-virus (HBV) and hepatitis C virus (HCV) have been the most common etiological factors in newly diagnosed HCCs in the last few decades, the introduction of viral vaccines and similar treatments has drastically reduced the incidence of viral-related HCC occurrence [2, 3]. In contrast, non-viral agents such as metabolic-associated fatty liver disease (MAFLD) have most recently become predominant drivers of HCC, being fueled by rising obesity and the associated comorbidities of type 2 diabetes mellitus (T2DM), as well as cardiovascular disease (CVD) [4–6]. Besides these triggering conditions, exposure to environmental toxins and chronic alcohol consumption represent other well-documented risk factors in the ability to prime the liver for the onset of HCC [7, 8].

Therapeutic options depend on the stage of the disease upon diagnosis, as early-stage HCC can either benefit from trans-arterial chemoembolization (TACE) or surgical resection [9]. Advanced-stage HCC is managed through liver transplantation or systemic chemotherapy [10]. The most commonly available therapeutic agents used generally encompass multi-kinase inhibitors (TKIs), including sorafenib, regorafenib, lenvatinib, cabozantinib and recombinant monoclonal antibodies, such as those targeting VEGF/VEGFR (bevacizumab, ramucirumab), or immune checkpoints (atezolizumab, nivolumab, and the above mentioned durvalumab and tremelimumab) [11]. Among the novel treatments currently being employed, atezolizumab + bevacizumab and durvalumab + tremelimumab are now recommended as the preferred first-line immune-based options for unresectable HCC. When administered in a randomized phase III trial (IMbrave150), atezolizumab + bevacizumab showed improvements in overall survival (OS) and progression-free survival (PFS); compared to sorafenib alone, the median overall survival was only about four months [12]. In another randomized phase III trial combining durvalumab + tremelimumab, OS improved when compared with sorafenib with no significant difference in FPS and only a marginal increase in median overall survival (< 3 months) [13].

Despite the entry of immune checkpoint inhibitors into clinical practice, multi-kinase inhibitors remain the standard-of-care drugs in HCC treatment. Sorafenib and its improved second-generation version, regorafenib, are being employed in patients with advanced HCC [14]. Regorafenib, which slows down angiogenesis and metastasis, has been correlated with more favorable patient outcomes, including overall survival, lower toxicity, and superior potential as a growth inhibitor [15]. Despite this wide spectrum of attack strategies, the clinical outcome of HCC remains unsatisfactory overall. A possible explanation may be found in the wide diversity of intratumor cell types involved, along with the multitude of intervening molecular factors that result in complex interactive scenarios. This is exacerbated by the occurrence of side effects often associated with treatments, especially those based on synthetic drugs.

A growing body of evidence highlights that a complex functional interplay occurs in HCC microenvironment, involving malignant hepatocytes, cancer-associated fibroblasts (CAFs), endothelial cells, infiltrating immune cells, growth factors, cytokines, chemokines, and extracellular matrix (ECM) molecules, ultimately affecting tumor growth and drug response [16–19].

Proteoglycans represent a diversified class of carbohydrate-conjugated proteins most often localized in the extracellular matrix, influencing multiple aspects of cell biology in solid tumors [20–22]. Proteoglycan-4 (PRG4) is a large mucin-like glycoprotein widely known for its lubricating and immune-modulating properties in joint tissues. It is also highly expressed in both in normal liver and in HCC [23, 24]. We have previously demonstrated that PRG4 is secreted by CAFs in HCC, which are one of the major sources of this proteoglycan, and it synergistically enhances the anti-proliferative effects of sorafenib and regorafenib on HCC cells in in vitro two-dimensional cell proliferation models. Furthermore, we established that the expression of cluster of differentiation 44 (CD44) on target HCC cells is essential for PRG4 to exert this drug-adjuvant activity [23, 25, 26]. CD44 was first characterized in leukocytes as a receptor for hyaluronic acid and is required by leukocyte homing in tissues following inflammation and immune activation. Besides physiological roles, CD44 was also described as a cancer stemness marker that may promote and support oncogenic processes like cell proliferation, migration, inflammation and angiogenesis, as well as endow cancer cells with drug resistance [27, 28]. More recently, PRG4 was discovered to bind CD44, and to trigger its downstream signaling in synoviocytes and fibroblasts in the context of normal joints and osteoarticular diseases [29]. While our findings pave the way toward the translational perspective of employing PRG4 as a therapeutic tool to ameliorate HCC treatment, they warrant further validation using more sophisticated experimental models beyond conventional assays. In this study, we investigated the synergistic anti-tumor effects of PRG4 and regorafenib in both in vivo orthotopic intrahepatic HCC xenografted tumors and in vitro three-dimensional tumorspheres.

## Methods

### Cells and reagents

The HLF cell line was purchased from JCRB Cell Bank (Japan). The HLC19 cell line is a primary spontaneously immortalized HCC cell line, used in a previous study [23]. All these cell lines were cultured in DMEM (Dulbecco’s Modified Eagle Medium) supplemented with sodium pyruvate, antibiotic-antimycotic, HEPES, and 10% fetal bovine serum (FBS) (Thermo Fisher Scientific). Cells were tested for the absence of mycoplasma contamination using the MycoFluor™ Mycoplasma Detection Kit (Thermo Fisher Scientific). Full-length recombinant human PRG4 (rhPRG4) was provided by Lubris Biopharma (Weston, MA, USA), and regorafenib (BAY 73–4506) was purchased from Cayman Chemicals (Ann Arbor, MI, USA). The antibodies used are reported in Suppl. Table 1.

### Stable PRG4 Expression

The construct used for the establishment of stable luciferase-expressing cell lines was designed and owned by Biogem S.c.ar.l. (AV, Italy). It consists of the Luc2 and Neo genes, derived from the pGL4.14 vector (Promega, WI, USA). The Luc2 coding sequence is regulated by the cytomegalovirus (CMV) immediate-early promoter, sourced from pcDNA3 (Invitrogen). To improve transcriptional stability and minimize position-effect variegation upon genomic integration, the entire expression cassette—comprising the CMV promoter, Luc2, and Neo—was flanked by H19 insulator sequences, originally obtained from the pWHERE vector. Standard molecular cloning techniques were employed for plasmid construction.

For the generation of stably transfected clones, the expression vector was linearized using NotI and transfected into target cells using Lipofectamine 3000 (Invitrogen), following the manufacturer’s protocol. Forty-eight hours post-transfection, the medium was replaced with a selection medium containing 400 µg/mL neomycin (G418, Gibco). Resistant cells were subsequently subjected to clone isolation by limiting dilution and then expanded in 96-well plates. Individual clones were evaluated using the Promega luciferase assay on an EnVision plate reader to assess transfection efficiency. Positive clones/pools were further expanded, analyzed via IVIS imaging, and cryopreserved at −80 °C.

HLC19 and HLF cell lines were transduced using lentiviral particles that contained either a control mock (empty) vector or a PRG4-expressing vector. Both vectors were purchased from Applied Biological Materials Inc. (abm, Richmond, BC, Canada), and were used according to the manufacturer’s instructions. One day before infection, cells were seeded at medium confluency. The next day (within 24 h), the medium was replaced, and lentiviral particles were added at a multiplicity of infection (MOI) 40 particles per cell in the presence of polybrene (8 µg/ml, Sigma-Aldrich/Merck, KGaA, Germany). After 2 days medium was replaced, and 4 days following infection selection with 1 µg/ml blasticidin (Thermo Fisher Scientific, Waltham, MA, USA) to obtain stable PRG4 expression was initiated. As soon as selection was complete, control and PRG4 expressing cells were screened for PRG4 expression and secretion by qPCR on total cell RNA, and western blot on cell conditioned medium. Control empty vector sequence and PRG4 expression vector were used in all the experiments.

### In vivo study

The in vivo studies were conducted at the Biogem S.c.a.r.l. (AV, Italy) animal facility. Cells from the cell lines of HLC19-Empty-LUC and HLC19-PRG4-LUC were intrahepatically injected into 20 CD1 nude female mice (1.5 × 10^6^ cells in 20 µl volume per inoculation). Tumor growth was measured at three time points through the IVIS Spectrum In Vivo Imaging System (Perkin Elmer, USA). On the fifth day, the mice were randomized into 4 experimental groups (of 10 animals each) and treated as follows:

**Table:** 

Experimental group	1	2	3	4
Injected cell line	HLC19-Empty-LUC	HLC19-Empty-LUC	HLC19-PRG4-LUC	HLC19-PRG4-LUC
Treatment	Vehicle 20 mg/ml DMSO, 1 v/v Kolliphor EL, in H_2_O	Regorafenib monohydrate CRS	Vehicle 20 mg/ml DMSO, 1 v/v Kolliphor EL, in H_2_O	Regorafenib monohydrate CRS

Administrations were carried out daily via gavage (OS, 10 mg/kg), starting from day 5 of the study up to day 34 or 13, depending on the study’s duration. All the animals were provided with complete feed and water *ad libitum*. Body weight for each animal was recorded weekly, starting from day 0 until the end of the study (Suppl. Figure 1 A). Tumor mass growth was assessed by means of an IVIS spectrum. Physical observations were conducted daily, including a visual check on the mice’s general appearance (e.g., fur and activity levels/responsiveness), and body weight for health status. Sacrifices were made on the day following the last administration by CO_2_, and tumor and liver samples were explanted. Tumor masses and the livers, where possible, were divided: one half was frozen at −80 °C and the second stored in 10% buffered formalin.

To evaluate longitudinal differences in IVIS imaging outcomes in both studies, two Generalized Estimating Equation (GEE) models were employed. These models were designed to account for intra-mouse variability arising from repeated measurements on the same animals and to robustly estimate the effects of treatment, cell line, and time [30–32].

In the first model, treatment–cell line combinations were entered as a four-level categorical factor (HLC19 PRG4 + Regorafenib, HLC19 Empty + Regorafenib, HLC19 PRG4 + Vehicle, and HLC19 Empty + Vehicle [control]), with time (in days) as a continuous variable and inclusion of an interaction term. The second model, restricted to the B76 IVIS outcome, considered both treatment–cell line combinations and time (8-day vs. 12-day [control, 8-day]) as two-level categorical factor.

### Western blotting

Mouse tissues were lysed with TissueLyser II (Qiagen^®^) and then resuspended using T-PER Tissue Protein Extraction Reagent supplemented with EDTA-free Halt Protease and Phosphatase Inhibitor Cocktail (Thermo Fisher Scientific). The proteins from HLC19 empty vector (EV) and PRG4 vector (PV) spheroids were extracted using the aforementioned solution. Lysates were incubated on ice for 30 min and vortexed every 10 min. The samples were then clarified through centrifugation at 17,010×g (at 4 °C) for 15 min to precipitate insoluble debris. The supernatants were assayed for protein concentration using Bradford Reagent (Bio-Rad). The proteins present in the conditioned media were utilized directly for the Bradford assay. The proteins were then mixed with Laemmli buffer and 10% β-mercaptoethanol (BME) and denatured at 95 °C for 5 min. 5 to 10 µg of total proteins were loaded onto a 4–20% Tris-Glycine eXtended (TGX) stain-free polyacrylamide gel membrane (Bio-Rad). After separation, proteins were transferred onto a polyvinylidene fluoride (PVDF) membrane (Trans-Blot^®^ Turbo Mini Format 0.2 μm PVDF, Bio-Rad) using the Trans-Blot Turbo Transfer System (Bio-Rad). The membrane was blocked for 1 h at room temperature with 5% non-fat dry milk in Tris-Buffered Saline with 0.05% Tween 20 (TBST), then incubated with primary antibodies, followed by horseradish peroxidase (HRP)-conjugated secondary antibodies. Signal detection was carried out using the Clarity Max Western ECL Substrate (Bio-Rad), and chemiluminescent signals were acquired with the ChemiDoc Imaging System (Bio-Rad).

### Eosin/hematoxylin staining

After sacrificing the mice, the tumors were excised and fixed in pH 6.8 to 7.2 buffered 10% formalin (Azer Scientific, Morgantown, PA, USA). The samples were than dehydrated using Diaphane (Histo-Line Laboratories, Milan, Italy) and Diawhite (DiaPath, Bergamo, Italy) alcoholic solutions, and finally embedded in paraffin (Merck, KGaA, Germany). 5 μm-thick tissue sections were cut, re-hydrated with Diaphane and Diawhite, and stained with eosin Y (Sigma-Aldrich/Merck, KGaA, Germany) and hematoxylin (Sigma-Aldrich/Merck, KGaA, Germany).

### Harvesting of conditioned medium

The four cell lines stably transfected with an empty control vector (EV) or PRG4 vector (PV) (HLC19-EV, HLC19-PV, HLF-EV, and HLF-PV) were seeded in a petri dish until confluence, washed and incubated in serum-free medium for another 48–72 h for secretome enrichment. The conditioned medium was then collected and concentrated using a Centricon^®^ device (3 kDa cutoff, Thermo Fisher Scientific).

### 2D proliferation assay

The proliferation assay was conducted as previously described on HLF and HLC19 cell lines. The four cell-lines (HLC19-EV, HLC19-PV, HLF-EV, and HLF-PV) were seeded in 100 µl of complete medium at 1 × 10^3^ seeding density in a 96-well plate and allowed to adhere overnight. After 24 h, the cells were pre-treated with their respective concentrated conditioned media (cCM) (100 µg/ml). At 48 h, designated as *t = 0*, the cells were divided into two groups. The first group, used as a control, was fixed with 4% paraformaldehyde (10-min incubation) and stained with crystal violet (CV), followed by thorough washing to remove excess dye. The second group was treated with either regorafenib (5 µM) or DMSO (vehicle control). After 72 h of treatment (*t = 72 h*), cells were fixed and stained as described above. Subsequently, 1% sodium dodecyl sulfate (SDS) was added to each well and left shaking until the complete release of CV from the cells. Optical density was then measured at 595 nm using a plate reader (Multiskan™ SkyHigh).

### 3D proliferation assay

HCC cell spheroids were generated by seeding 1 × 10^3^ cells per well of the four cell lines in a 96-well ultra-low attachment round bottom plate. Once they assumed a characteristic spheroid shape (after 4 days), spheroids were embedded in Matrigel^®^, allowed to settle for 3 days, and then treated with either DMSO as a control (vehicle) or regorafenib (5 µM) for four days (t = 96 h), with a medium and treatment renewal after 48 h. The spheroids were then photographed with the Eclipse Ti2 microscope (Nikon Corp., Japan) and their area was measured at both at t = 0 h and at t = 96 h. Growth was calculated as the incremental percentage difference of area compared between t = 0 h and at t = 96 h.

### Cell cycle assay

Spheroids made by 1 × 10^4^ HLC19-EV and HLC19-PV cells were fabricated as previously described. After 4 days of culture, 24 spheroids per well were embedded in 300 µl of pre-chilled Matrigel and layered onto 400 µl of pre-polymerized Matrigel in a 12-well plate. Following polymerization (~ 20 min), 1.5 mL of complete DMEM was added, and the plate was cultured for 3 days. The medium was then replaced and treatment with DMSO (vehicle) or regorafenib (5 µM) was initiated. Treatments were renewed after 48 h and continued for a total of 4 days. At the endpoint, the medium was removed and 2 ml of a dissolution buffer made by 1X Tryple Select (Gibco, Thermo Fisher Scientific, USA) + 1 mM EDTA in PBS was added to dissolve the Matrigel and dissociate cells. After pipetting and incubation at 37 °C, an additional 8 ml of dissolution buffer was added. This mixture was centrifuged at 600×g for 7 min to pellet cells, which were resuspended in 500 µl of cold PBS. 1.17 ml of pre-chilled absolute ethanol was added to the pellet while gently vortexing (to reach 70% ethanol concentration) to fix and permeabilize cells. After the fix/permeabilization step (2 h at 4 °C) cells were centrifuged at 800×g for 7 min and washed twice (by centrifuging and supernatant discarding) with a permeabilization buffer (PB: PBS + 0.1% Triton-X100). An RNA degradation step was then performed by incubating cells with permeabilization buffer containing RNase A (100 µg/ml) at 37 °C for 1.5 h. Cells were finally washed once, resuspended in 500 µl of PB containing propidium iodide (PI, 25 µg/ml), and analyzed for cell cycle distribution using the Navios flow-cytometer (Beckman-Coulter, USA) for data acquisition and the proprietary Kaluza Analysis Software version 1.5a for quantification.

### Immunofluorescence

Tumorspheres fabricated as previously described before were fixed with 4% PFA for 30 min at room temperature and then washed with 0.1% Tween-20 in PBS. They were then permeabilized with a washing buffer (0.1% Tween-20 and 0.2% BSA in PBS) for 30 min at 4 °C. The samples were stained for primary antibodies (Table **Z**) and incubated overnight at 4 °C. The following day, post-washing, the samples were incubated with secondary antibodies and DAPI for 1 h at room temperature. After another round of washing, the samples were centrifuged at 300×g for 3 min and resuspended in a clearing solution (2.5 M fructose in 60% glycerol). They were then placed on a covered glass slide, incubated for 2 h at 4 °C and visualized through an Eclipse Ti2 Confocal Microscope (Nikon Corp., Japan).

### RNA extraction and cDNA synthesis

Post treatment, spheroids were subjected to lysis using the TRIzol™ Reagent (Qiagen, USA) and RNA was extracted as per the manufacturer’s instructions (Thermo Fisher Scientific, USA). The quality and concentration of isolated RNA were measured using the NanoDrop 2000/2000c (Thermo Fisher Scientific, USA). Contaminating DNA was removed from RNA by using the Turbo DNA-*free* kit (Ambion, Thermo Fisher Scientific, USA). cDNA was synthesized using the High-Capacity cDNA Reverse Transcription Kit (Thermo Fisher Scientific, USA) as per instructions.

### Real-time polymerase chain reaction (qRT-PCR)

1 ng/µl of cDNA was loaded into 20 µl total reaction mix in the presence of 500 nM each of forward and reverse primers specific to the gene of interest, along with 2× SYBR Green Master Mix (Bio-Rad, USA). The reaction was conducted in a CFX96 Touch Real-Time Detection System (Bio-Rad, USA). More information on primers used is reported in Suppl. Table 2.

### Whole transcriptome profiling

Total RNA samples were reverse transcribed using the Ion Torrent™ NGS Reverse Transcription Kit (Thermo Fisher Scientific, Waltham, MA, USA) in accordance with the manufacturer’s instructions. Target region amplification was performed using the Ion AmpliSeq Transcriptome Human Gene Expression core panel (Thermo Fisher Scientific, Waltham, MA, USA) on the Ion Chef System. The barcoded libraries were quantified by qPCR with the Ion Library TaqMan Quantitation Kit (Thermo Fisher Scientific, Waltham, MA, USA). Finally, libraries were templated onto the Ion Chef and sequenced using a 540 chip on the Ion GeneStudio S5 Prime system (Thermo Fisher Scientific, Waltham, MA, USA). The transcriptomic data generated in this study have been deposited in the GEO repository and are publicly available under the accession number GSE299840 (https://www.ncbi.nlm.nih.gov/geo/query/acc.cgi?acc=GSE299840).

### Bioinformatics and statistical analyses

Raw read counts related to transcription data were generated using the ampliSeqRNA plugin of the Ion Torrent Suite Server v5.16.1 (Thermo Fisher Scientific, Waltham, MA, USA) with default settings. Downstream analyses were performed using the Transcriptome Analysis Console 4.0 software (Thermo Fisher Scientific, Waltham, MA, USA). DEGs were identified with the Limma eBayes method using a 1.5 threshold of fold-change and p-value ≤ 0.05. Alt Analyze 2.1.3 software was used to generate hierarchical clustering [33]. Biological processes (signaling pathways and molecular networks) associated with DEGs were analyzed using Ingenuity Pathway Analysis (IPA) software (Qiagen, USA). Biological and technical replicates of in vitro studies were analyzed using the Student’s T-test (unpaired, two-tailed).

GEE models were fitted using either Gaussian or Gamma distributions with an identity link function, depending on the distributional characteristics of the outcome variables. An exchangeable correlation structure and a robust (sandwich) variance estimator were applied to correct for potential model misspecifications. Model parameters associated with time (β_T_) were interpreted as average daily changes in the IVIS signal, those associated with treatment (β_TR_) were interpreted as average differences between treatments at the first post-baseline measurement, and the interaction parameters (β_T×TR_) were interpreted as average differences in time-dependent changes between treatment groups.

Post hoc multiple comparison analyses were subsequently performed to identify statistically significant differences between factor levels within each model. In addition, regarding angiogenesis array (see next paragraph) comparisons between membrane types (PRG4 vs. Empty Vector cCMs) at 48 and 72 h were conducted using the Mann–Whitney U test, with protein measurements treated as independent statistical units.

All statistical analyses were performed using GraphPad Prism 10.0 (Boston, MA, USA) and R software (Vienna, Austria) with the packages geepack (for GEE fitting), lsmeans, and emmeans (for post hoc analyses) [31, 34–38]. A p-value ≤ 0.05 was considered statistically significant.

### Angiogenesis array

The expression of angiogenesis-related proteins was evaluated using the Proteome Profiler Human Angiogenesis Array Kit (ARY007, R&D Systems, Minneapolis, MN, USA), following the manufacturer’s instructions. 40 µg of HLC19 EV and PV cCMs were incubated with a cocktail of biotinylated detection antibodies and applied to nitrocellulose membranes spotted in duplicate with capture antibodies. Bound proteins were detected using streptavidin-HRP and chemiluminescent substrate. Signals were visualized by chemiluminescence imaging and quantified by densitometric analysis (ImageJ, plug-in Protein Array Analyzer), with background subtraction and normalization to internal positive and negative controls.

### Tubulogenesis assay

40 µl of Matrigel was layered onto wells on a 96-well plate and left to polymerize for 20 min. 100 µl of complete endothelial cell medium (EndoGro, Millipore/Merck, KGaA, Germany) was then added to each well to pre-condition the Matrigel and removed after 1 h. 1.7 × 10^4^ of HUVEC cells (Millipore/Merck, KGaA, Germany) were resuspended in 100 µl of medium in the presence of concentrated Conditioned Medium (cCM) of PRG4-PV and PRG4-EV HLC19 cells and/or regorafenib, and seeded onto Matrigel. The final protein concentration of cCM and regorafenib were 200 µg/ml and 2.5 µM, respectively. These concentrations were adjusted (increased by 1.4 times) in accordance with the presence of the Matrigel base at the bottom of the well, to reach the final nominal concentrations. DMSO and serum-free DMEM were used as vehicles for regorafenib and cCM, respectively. Tube formation was monitored for 14 h using time-lapse imaging at 37 °C and 5% CO₂. Image acquisition for each condition began at time zero and was performed every 10 min, capturing one field (40× magnification) per well. Each treatment was performed in triplicate.

## Results

### Intratumor PRG4 overexpression decreases tumor development and enhances regorafenib counter-proliferative effects in an orthotopic intrahepatic HCC model

**Fig. 1:**
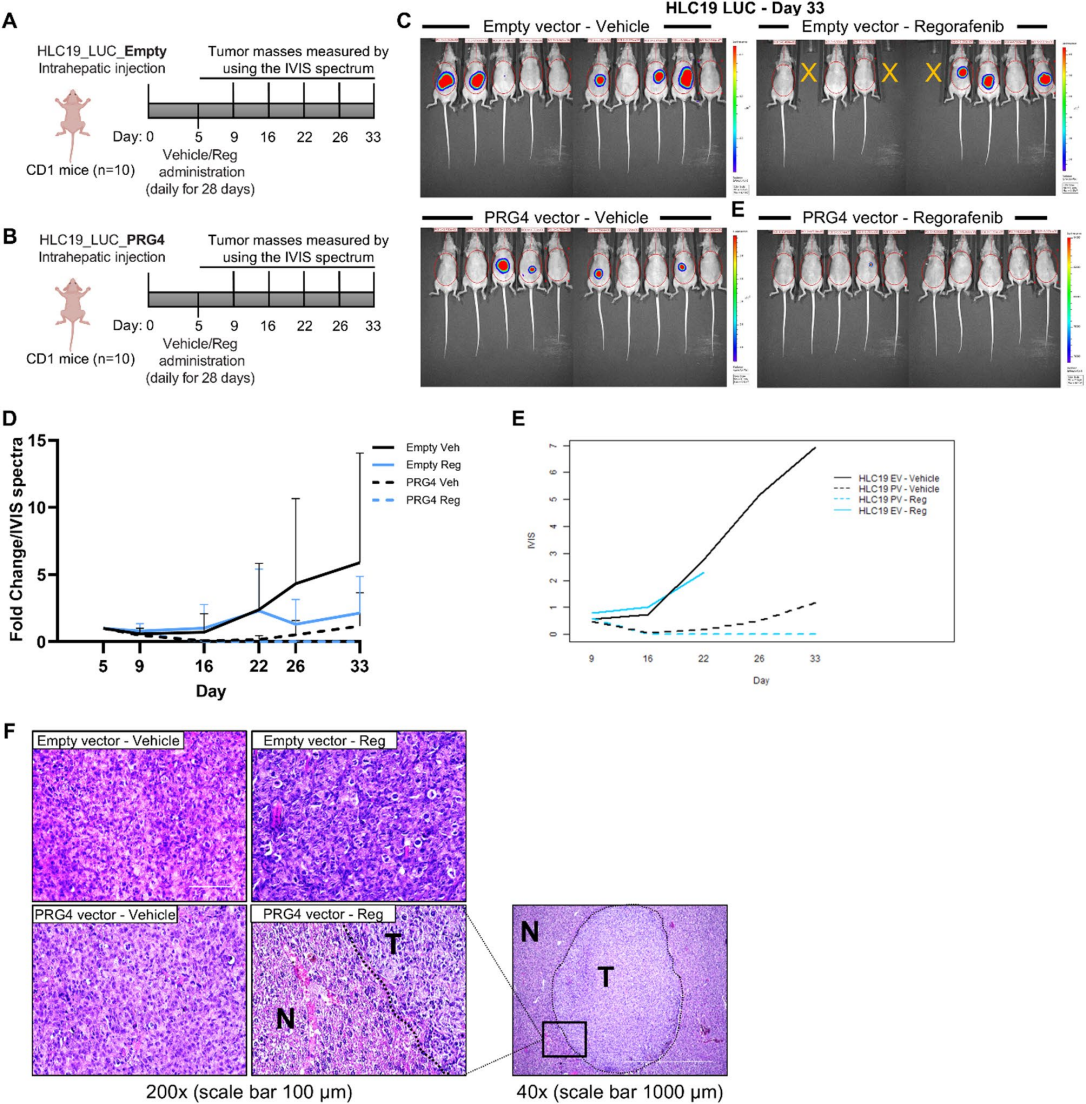
PRG4 enhances antiproliferative effect of regorafenib in an in vivo xenograft orthotopic intrahepatic HCC model. **A**-**B** Experimental design of the orthotopic murine HCC model (B56 study). Forty nude female mice of CD1 strain were randomized and divided into 2 groups of 20 units each, and intrahepatically injected with HLC19 LUC EMPTY (PRG4^−^) lentiviral vector, or HLC19 LUC PRG4 (PRG4^+^) lentiviral vector cells. On the fifth day, the animals of either group were randomized and further divided into 2 treatment groups, receiving either regorafenib (10 mg/kg) or vehicle, daily for 29 days. Tumor progression was monitored using IVIS Spectrum imaging on days 5, 9, 16, 22, 26, and 33. The sacrifice was made on the 35th day. **C** IVIS images at day 33. Areas of tumors are circled in red. Signal intensity is shown as a heatmap, wherein red denotes high and blue low luminescence. Yellow crosses mark animals sacrificed before day 33 due to signs of suffering/metabolic distress. **D** Quantification of IVIS signal over time for each group. Data are presented as mean ± SD. Black solid line: HLC19-EV + vehicle; black dashed line: HLC19-PV + vehicle; blue solid line: HLC19-EV + vehicle; blue dashed line: HLC19-PV + regorafenib. The tumor growth over time was normalized with respect to the signal detected on day 5 (= 1) for each mouse. **E** Interaction analysis to assess synergism between PRG4 and regorafenib in the orthotopic HCC model. **F** Representative hematoxylin and eosin (H&E) staining images of tumor tissues from each group, showing microscopic histological morphology. Note that the lower right image refers to the sole animal of the PRG4^+^ + regorafenib cohort for which it was possible to instrumentally detect the presence of a mass (shown at lower magnification in the rightmost image). T: tumor; N: normal liver

HLC19 cells were engineered to stably express luciferase along with either a lentiviral control vector (PRG4^−^) or a PRG4 overexpressing vector (PRG4^+^). These cells were then intra-hepatically injected into nude CD1 mice (*n = 20* per group) to produce experimental HCC tumors. Each cohort was further randomized into two treatment groups (*n = 10*), treated with either vehicle or regorafenib (10 mg/kg, Fig. 1A, B). As seen from IVIS imaging and following quantification of tumor progression **(**Fig. 1C, D**)**, PRG4^+^ tumors of vehicle-treated animals exhibited an overall delayed growth when as compared to PRG4^−^ tumors of vehicle-treated animals, although the difference was statistically significant only at day 22 post-injection. This result is consistent with the positive correlation found in human HCC tumor samples between the mRNA expression of PRG4 and the overall survival of patients (Suppl. Figure 2). Even though regorafenib limited the growth of PRG4^−^ tumors compared to the vehicle, the differences were not significant. In contrast, regorafenib markedly suppressed the growth of PRG4^+^ tumors at all time points of IVIS detection (16th, 22nd, 26th, and 33rd days), with statistically significant differences compared to regorafenib-treated PRG4^−^ tumors, as determined by the Mann-Whitney U test (Table 1). Notably, regorafenib treatment induced a near-complete tumor regression in the PRG4^+^ cohort, as by day 33 only one PRG4^+^ mouse had a detectable tumor mass of ∼1.5 mm as confirmed by IVIS imaging and subsequent histological analysis (Fig. 1C, F). An analysis to evaluate whether the interaction between PRG4 and regorafenib is synergistic or additive was also performed. The interaction plots across days of the IVIS capture values in relation to the ‘cell line-treatment combination’ demonstrate a synergistic, rather than additive, interaction between PRG4 and regorafenib (Fig. 1E). Details of interaction analysis are shown in Suppl. Statistical data.

**Table 1:** Mann–Whitney *U* test applied to the orthotopic murine HCC model (B56 study) comparing treatment effects across groups and treatment arms

Mann-Whitney U Test		Day			
		16	22	26	33
Empty vector	Veh - Reg	0.8493	0.7949	0.1211	0.1868
PRG4 vector	Veh - Reg	0.0891	0.0316*	0.0114*	0.0013*
Empty vector - PRG4 vector	Veh - Veh	0.0751	0.0316*	0.0536	0.1211
	Reg - Reg	0.0013*	0.0006*	0.0147*	0.0029*

*P*-values marked with an asterisk refer to statistically significant differences (*p *<0.05)

**Fig. 2:**
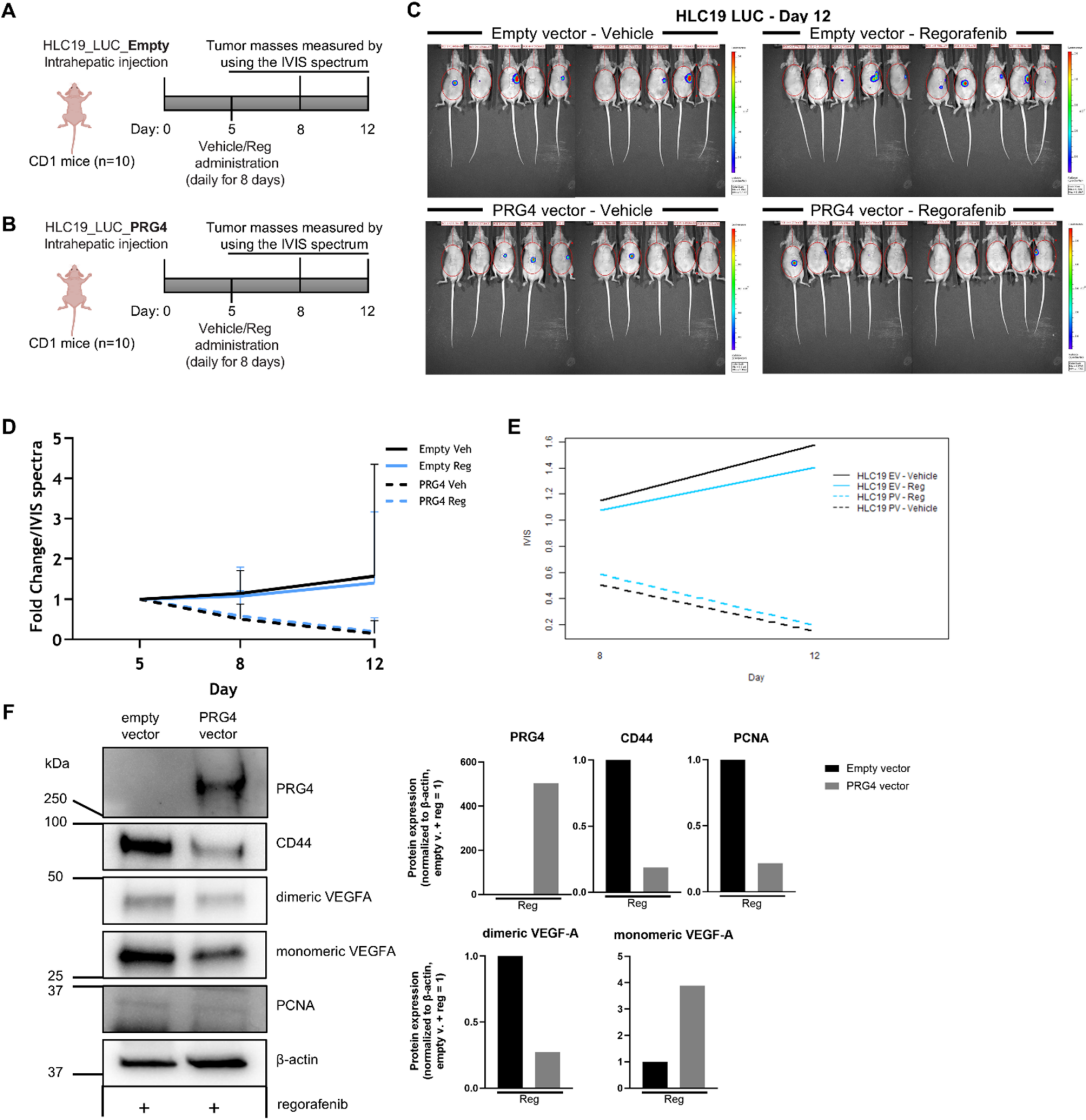
PRG4 amplifies the regorafenib-induced downregulation of proliferation and angiogenic markers in HCCin vivo. **A**-**B** Experimental design for the short-term replication of the orthotopic mouse model of HCC (B76 study). A total of 40 mice were randomized, intrahepatically inoculated, and treated as in the previous study (Fig. 1) except for the overall shorter duration (14 days) before sacrifice. **C** IVIS images from day 12. Tumor regions are circled in red. Signal intensity is shown as a heatmap, where red denotes high and blue low luminescence. **D** Quantification of IVIS signal over time for each group. Data are presented as mean ± SD. Black solid line: HLC19-EV + vehicle; black dashed line: HLC19-PV + vehicle; blue solid line: HLC19-EV + regorafenib; blue dashed line: HLC19-PV + regorafenib. **E** Interaction analysis to assess synergism between PRG4 and regorafenib in the orthotopic HCC model. **F**, left Western blot analysis of protein lysates from two tumor tissue related to Empty vector + regorafenib and PRG4 vector + regorafenib, showing the greater inhibition of the proliferation marker PCNA and of the pro-angiogenetic ligand VEGF as a result of combined PRG4 + regorafenib treatment than regorafenib treatment alone. **F**, right Densitometric analysis of western blot: protein levels were normalized to β-actin as a housekeeping control, and then to Empty vector + regorafenib (= 1). Images of livers containing the tumor masses explanted for western blot analysis of proteins shown in panel F, are reported in Suppl. Figure 1B. The western blots pertaining to all the tumor samples collected from B56 and B76 studies is shown in Suppl. Figure 3. Full blot images for F panel are provided in the Suppl. Figure 4

Due to dramatic tumor regression and the virtual absence of masses in the PRG4^+^ + regorafenib group, although some tissue samples were collected at the conclusion of the study, they were not sufficient in number for a deepen downstream molecular analysis. Therefore, in an attempt to obtain resectable tumor tissues before regression and gain mechanistic insights into the PRG4 and regorafenib synergy, a replicate experiment was conducted with animals being sacrificed at day 12 when tumor size became significant (*p* < 0.05) (Fig. 2A-E**)**. Statistical significance of differences among treatments/cohorts in this in vivo experiment was detected by performing the Mann-Whitney U test (Table 2). Although the growth of regorafenib + PRG4^+^ tumors was not completely inhibited, unlike in the first experiment, only one residual nodule was detected in the same group **(**Fig. 2C**)**. We then compared the expression of key proteins related to proliferation and angiogenesis in this nodule with that of a tumor from the regorafenib-only treatment **(**Fig. 2F, left). Densitometric analysis **(**Fig. 2F, right) showed a reduced expression of proliferating cell nuclear antigen (PCNA), as well as vascular endothelial growth factor A (VEGFA, both monomeric and dimeric) in the regorafenib + PRG4^+^ group. This suggests that PRG4 enhances regorafenib’s antiproliferative and anti-angiogenic activity. Interestingly, CD44 expression was strongly downregulated in the PRG4 + regorafenib group compared to the regorafenib-only group. The downregulation of CD44 expression under the latter treatment conditions suggests that a loss of cancer stemness traits in tumors may partially explain the observed failure in the establishment and/or development of nodules. The expression levels of PRG4, CD44, and VEGF-A in the PRG4^+^ tumors were compared to their PRG4^−^ counterparts from both untreated and regorafenib-treated groups. This comparison was conducted after the excision of tumor masses from animals that were sacrificed at the conclusion of studies B56 and B76, and the results were confirmed via qPCR (Suppl. Figure 5).

**Table 2:** Mann–Whitney *U* test applied to the orthotopic murine HCC model (B76 study) comparing treatment effects across groups and treatment arms

Mann-Whitney U Test		Day	
		8	12
Empty vector	Veh - Reg	0.4295	0.6745
PRG4 vector	Veh - Reg	0.9681	0.6241
Empty vector - PRG4 vector	Veh - Veh	0.0091*	0.0891
	Reg - Reg	0.0257*	0.0455*

*P*-values marked with an asterisk refer to statistically significant differences (*p *<0.05)

### PRG4 increases regorafenib antiproliferative effects on HCC cells in 2D in vitro models

**Fig. 3:**
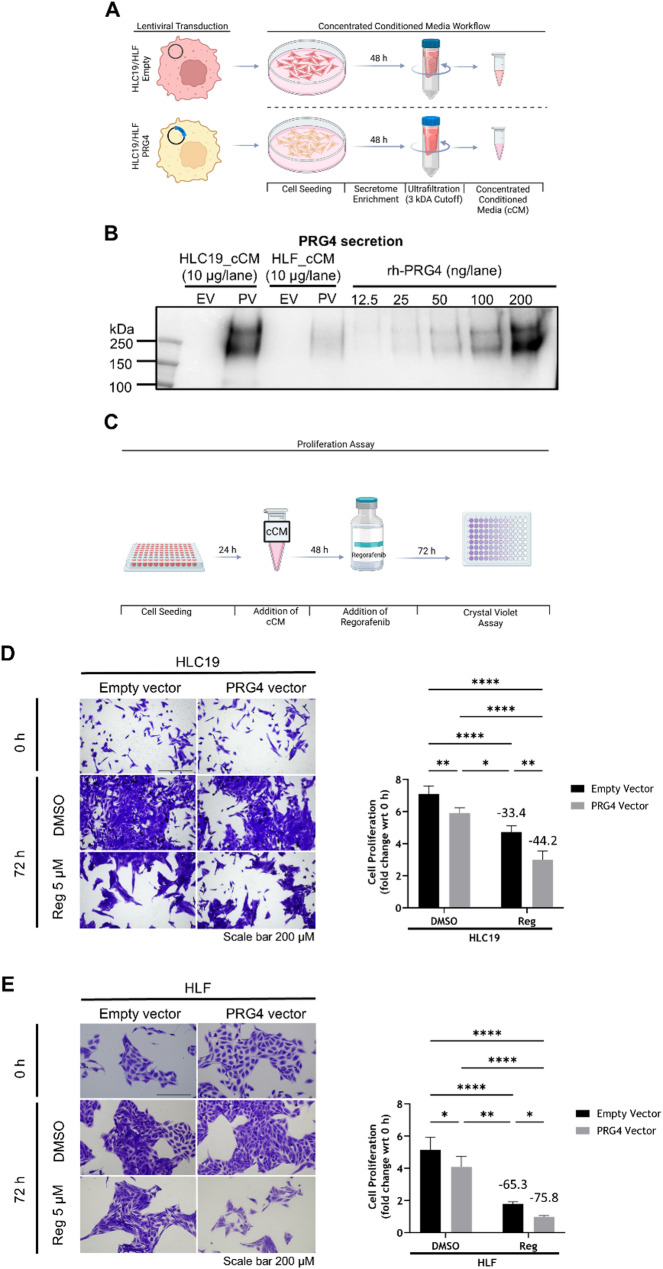
HCC cell self-secreted PRG4 synergizes with regorafenib in inhibiting cell proliferation in an in vitro 2D culture model. **A** Schematic protocol to obtain concentrated Conditioned Medium (cCM) from HLC19 and HLF cell lines transfected with lentiviral empty vector and PRG4 vector. The different steps are shown. **B** Western blotting to estimate the amount of secreted PRG4 in reference to a titration curve using known quantities of human recombinant full-length PRG4 (rh-PRG4). **C** Schematic protocol of 2D in vitro proliferation assay. **D**,** E**, left Representative images of crystal violet stained HLC19 and HLF cells illustrating the differences in cell proliferation across conditions. **D**,** E**, right Quantification of cell proliferation upon crystal violet dissolution. Bars represent mean ± SD of experiments repeated in triplicate. The numbers above histograms represent the percent inhibition of cell proliferation in control (empty vector) and PRG4-expressing cells by regorafenib compared to DMSO-treated counterparts. Statistical analysis was performed using two-way ANOVA test. Differences were considered statistically significant for *p* < 0.05 (*: *p* < 0.05; **: *p* < 0.01; ****: *p* < 0.0001)

To validate the in vivo findings in a less complex 2D HCC cell growth model, cell proliferation assays were performed using the same HCC cell lines used in in vivo experiments (HLC19 EV/PV), and an additional HCC cell line, HLF engineered in the same way (HLF EV/PV) to assess PRG4 + regorafenib synergism. It should be noted that the transduction of HCC cells with the PRG4 gene-carrying vector does not affect HCC cell proliferation by itself (Suppl. Figure 6 A, B). As PRG4 is a secreted proteoglycan that acts in an autocrine/paracrine manner by binding with CD44, its sufficient accumulation in the extracellular milieu is necessary to exert its biological effects. However, in low-density cell cultures, this accumulation may not reach sufficient concentrations within the time frame of the proliferation assays. To overcome this limitation, we first collected and concentrated total proteins derived from serum-free conditioned medium of HLC19 and HLF EV/PV after 48 h of culture (cCM) **(**Fig. 3A**)**. PRG4 content in the cCM was determined through western blotting and compared with recombinant human PRG4 (rhPRG4) as a reference. PRG4 secreted by HLC19 represented ~ 2% of total protein (w/w) while that secreted by HLF was 4 times lower, at roughly ~ 0.5% of total protein (Fig. 3B). These cCM were then used to precondition the respective cells of origin (100 µg/ml total cCM protein) for 48 h. Subsequently, the cells were exposed to DMSO (vehicle) or regorafenib (5 µM) for 72 h to finally evaluate cell proliferation through a crystal violet dissolution assay (Fig. 3C). Unsurprisingly, regorafenib reduced cell proliferation in HLC19-EV and HLF-EV cells when compared to DMSO. This effect was further amplified in PRG4-expressing cells, which suggested a synergistic antiproliferative effect of PRG4 and regorafenib, as established in the xenograft model. Synergistic effect was assessed by an interaction two-way ANOVA test. Although a synergism (i.e. interaction) occurred between PRG4 and regorafenib, the relative statistical tests were not significant for either HLC19 cells (null hypothesis: interaction = 0, *p* = 0.3227 > 0.05, additivity), and HLF cell line (null hypothesis: interaction = 0, *p* = 0.6620 > 0.05, additivity). (Fig. 3D, E). The expression and production of PRG4 in HLC19 and HLF EV/PV cells was determined by visualizing (by immunofluorescence) cell culture plates coated with secreted PRG4, and quantifying PRG4 mRNA (by qPCR) (Suppl. Figure 6 C, D).

### PRG4 amplifies the antiproliferative effects of regorafenib on HCC cells in 3D in vitro models

**Fig. 4:**
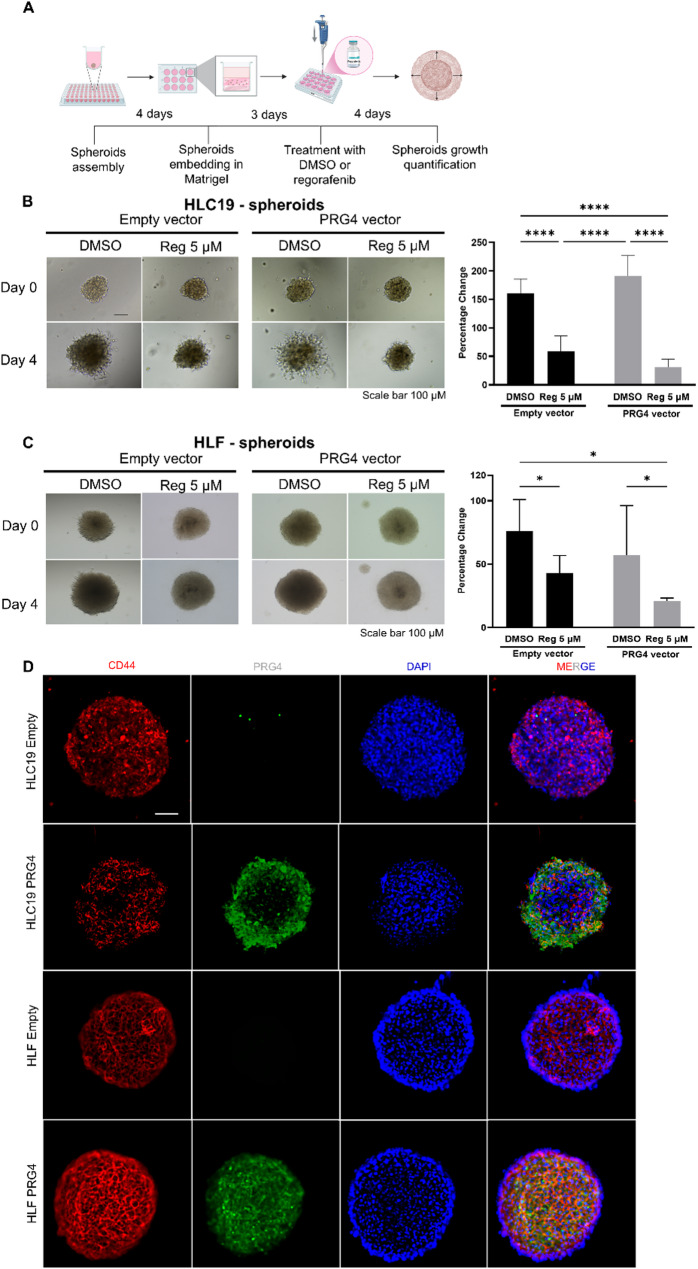
PRG4 potentiates the effect of regorafenib in delaying the expansion of 3D Matrigel embedded HCC tumorspheres. A Graphical representation of the method used for the generation and treatment of the HCC spheroids. **B**,** C**, left Pre-assembled tumorspheres made by empty vector or PRG4 vector HLC19 and HLF cells were Matrigel-embedded and treated with 5 µM regorafenib or DMSO for 4 days. Spheroids were photographed and their area measured at day 0 and 4 of treatment (scale bar: 100 μm). **B-C**,right Quantification of percentage change of growth referred to Day 0. Bars represent mean ± SD of the percentage change of spheroids area (*n* = 7–10 for HLC19; *n* = 4–6 for HLF). Statistical analysis was performed using 2way Anova test. Differences were considered statistically significant for *p* < 0.05 (*: *p* < 0.05; ****: *p* < 0.0001). **D** Immunofluorescence staining of pre-assembled floating (non-Matrigel-embedded) Empty vector or PRG4 vector tumorspheres. Spheroids were stained for CD44 (red), PRG4 (green), and nuclei were counterstained with DAPI (blue). Merged images show co-localization of markers. Scale bar: 100 μm

To support the in vivo findings, a 3D spheroid model was employed as it closely recapitulates the architectural features of a tumoral microenvironment, like the presence of a surrounding extracellular matrix (ECM) and nutrient diffusion gradients. HLC19 and HLF EV and PV cell spheroids were allowed to form, then embedded in Matrigel, and treated with either DMSO or regorafenib (5 µM) for four days (Fig. 4A). Consistent with the 2D assays, regorafenib significantly delayed the growth of spheroids, which was measured as percentage increase in area relative to day 0, in both cell lines. Notably, the presence of PRG4 significantly enhanced the drug’s inhibitory effect under 3D conditions. Synergistic effect was assessed by an interaction two-way ANOVA test. Although a synergism (i.e. interaction) occurred between PRG4 and regorafenib, the relative statistical tests were significant only for HLC19 cells (null hypothesis: interaction = 0, *p* = 0.0023 < 0.05, no parallelism), but not for HLF cell line (null hypothesis: interaction = 0, *p* = 0.8947 > 0.05, additivity). (Fig. 4B and C). The presence of PRG4 exclusively in spheroids formed by PRG4-expressing cells from both cell lines was evidenced by immunofluorescence analysis on Matrigel-free spheroids **(**Fig. 4D**)**.

### PRG4 extends regorafenib-induced cell cycle delay in 3D spheroids

**Fig. 5:**
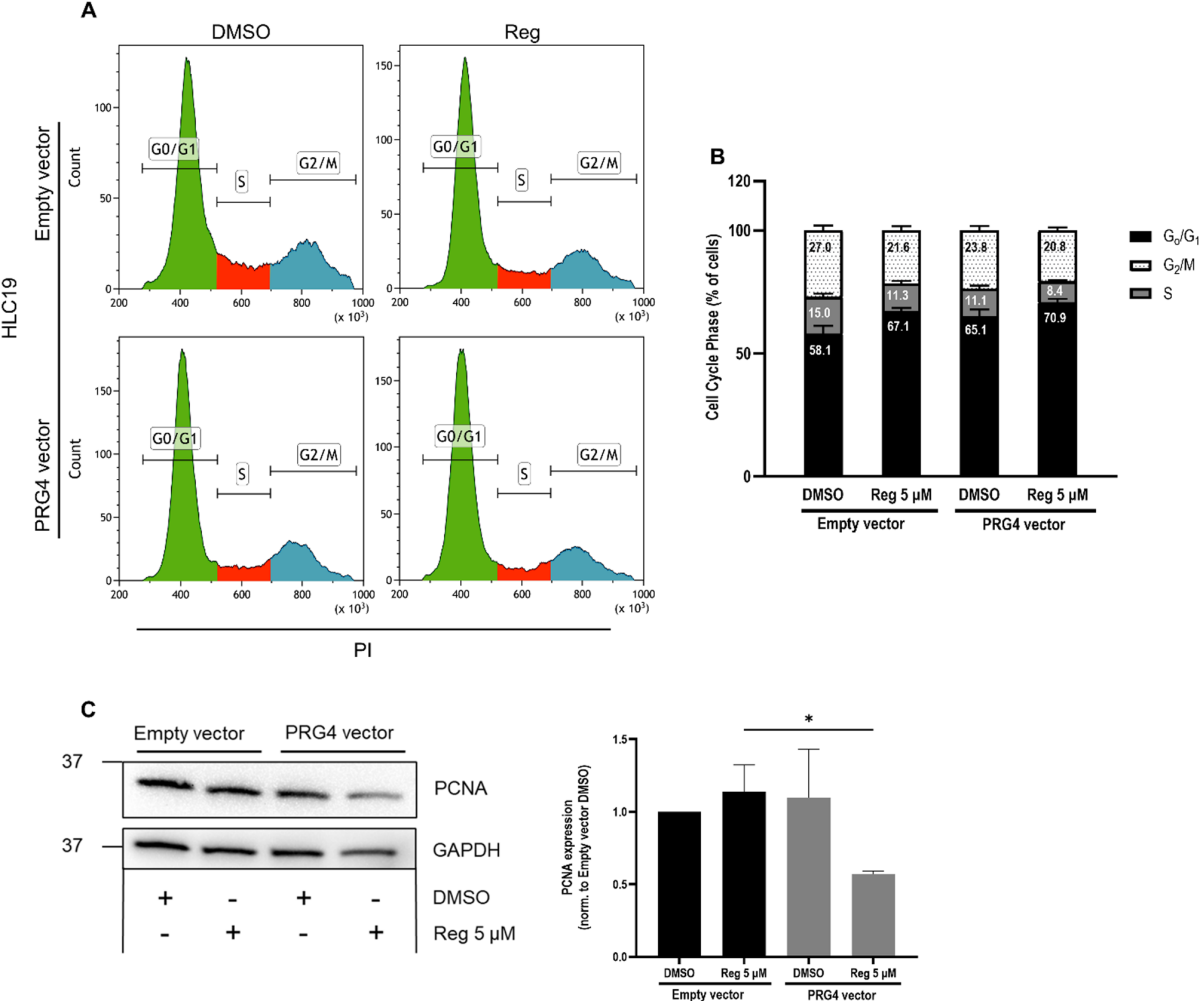
PRG4 extends regorafenib-induced delays of cell cycle progression in Matrigel-embedded HCC cell tumorspheres. **A** Flow cytometric analysis of cell cycle phase distribution was conducted in Empty vector and PRG4 vector HLC19 cell-derived spheroids treated with DMSO and 5 µM regorafenib, as in the Fig. 4 (A-B). **B** Data were analyzed using a stacked column chart displaying the percentage distribution of cells in G_0_/G_1_, S, and G_2_/M phases of cell cycle. Results are expressed as mean ± SD shown are from three independent experiments. **C**, left Western blotting showing the expression of PCNA in HCL19 Empty vector and PRG4 vector spheroids treated in the same experimental condition used for cell cycle analysis. GAPDH was used as a housekeeping gene for normalization. **C**, right PCNA band intensities were densitometrically quantified and normalized first to GAPDH, and then to Empty vector + DMSO condition (= 1). Whole blots are shown in Suppl. Figure 7. Results are expressed as mean ± SD shown are from three independent experiments. Statistical analysis was performed using t-test (unpaired, two-tailed); *: *p* < 0.05

An analysis of cell cycle phase distribution was performed to assess the PRG4-driven enforcement of regorafenib’s antiproliferative effect in Matrigel-embedded spheroids. HLC19 spheroids treated with either DMSO or regorafenib as described in the 3D growth assay (Fig. 4), were disaggregated to obtain single-cell suspension. Flow cytometry was then used to quantify DNA content through the detection of intercalated propidium iodide (PI), which was correlated with cell cycle phase. As expected, in PRG4^−^ HLC19 spheroids, the percentage of cells in G_0_/G_1_ increased as a result of the regorafenib treatment. Most importantly, PRG4 expression not only increased the proportion of cells in the G_0_/G_1_ phase, but also further delayed the exit of cells from this phase in the presence of regorafenib (Fig. 5A, B**)**. The cell cycle statistical analysis is reported in Table 3. The expression of Proliferating Cell Nuclear Antigen (PCNA) estimated through western blotting in cells derived from spheroids treated as above resulted in significant diminution in regorafenib-treated PRG4^+^ spheroids, to a greater extent than in regorafenib-treated PRG4^−^ spheres, further corroborating the synergistic interplay between PRG4 and regorafenib in limiting the expansion of spheroids (Fig. 5C).

**Table 3:** T-test (unpaired, two-tailed) for the comparison of the effects of experimental conditions in the phases of the cell cycle and in treatment groups

		Empty vector	PRG4 vector	
		Reg 5 µM	DMSO	Reg 5 µM
**G**_0_ **/G**_1_				
Empty vector	DMSO	0.0103*	0.0431*	0.0029*
	Reg 5 µM		0.3237	0.0254*
PRG4 vector	DMSO			0.0308*
**S**				
Empty vector	DMSO	0.0163*	0.0204*	0.0010*
	Reg 5 µM		0.8272	0.0091*
PRG4 vector	DMSO			0.0270*
**G**_2_ **/M**				
Empty vector	DMSO	0.0202*	0.0953	0.0085*
	Reg 5 µM		0.1727	0.5012
PRG4 vector	DMSO			0.0593*

P-values marked with an asterisk refer to statistically significant differences (p <0.05)

### PRG4 restrains in vitro endothelial tubulogenesis

**Fig. 6:**
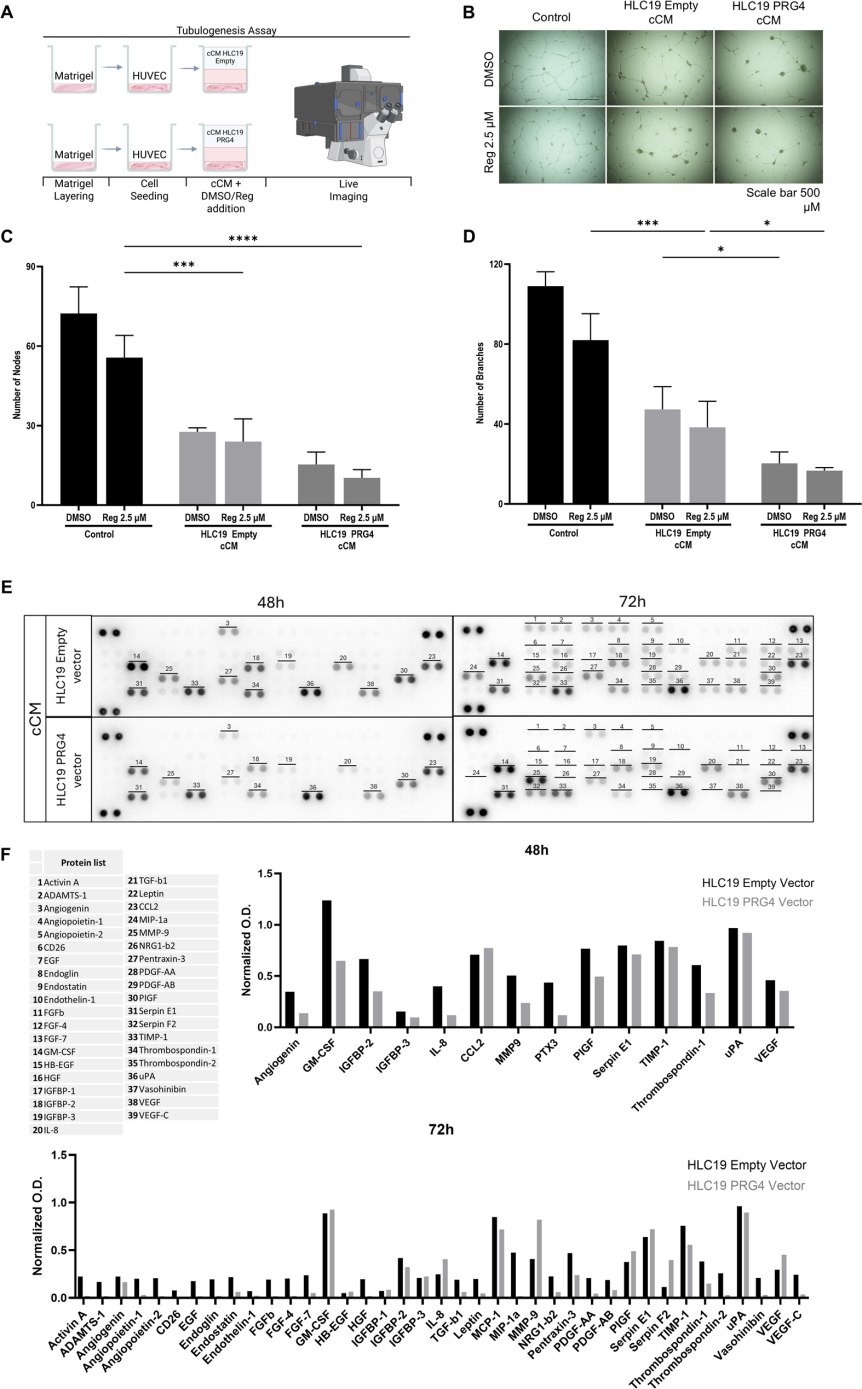
PRG4 impairs endothelial angiogenesis in vitro independently of regorafenib. **A** Schematic protocol of angiogenetic assay. HUVEC cells were layered onto a Matrigel substrate and exposed to cCM (at a concentration of 200 µg/ml) from Empty vector and PRG4 vector HLC19 cells, in the presence of DMSO or 2.5 µM regorafenib and network formation was monitored up to 14 h. **B** Representative images of tubular endothelial structures at 14 h of treatment. **C**, **D** Quantification of number mature nodes and branches as indexes of proficiency of HUVEC cells in perform tubulogenesis under the tested treatment conditions after 14 h. Results are expressed as mean ± SD shown are from experiments performed in triplicate. For statistical analysis t-test was applied (unpaired, two-tailed); *: *p* < 0.05; ***: *p* < 0.001. **E** Profile of angiogenesis-related protein factors in cCM of HLC19-EV and HLC19-PV cells, collected after 48 and 72 h hours. Each factor is detected as a spot in duplicate. **F** List of angiogenesis-related factors detected in cCMs of HLC19-EV and HLC19-PV cells and related densitometry quantification graphs

The evidence of VEGF downregulation in response to PRG4 expression within xenografted tumors prompted us to further investigate the putative anti-angiogenic role of this proteoglycan, either alone or in combination with regorafenib, to enhance its inhibitory effect on angiogenesis. We performed an in vitro tubulogenesis assay to evaluate the ability of endothelial cells (HUVEC) to undergo tubulogenesis when exposed to concentrated conditioned medium (cCM) from a 48-hour enriched secretome of HLC19 EV/PV cells. This was done both individually and in combination with regorafenib (Fig. 3). Once seeded onto a matrigel scaffold, these cells were exposed to cCM (200 µg/ml total protein concentration) derived from HLC19 EV/PV cells in the presence/absence of regorafenib (2.5 µM) (Fig. 6A). After 14 h, regorafenib induced a slight inhibition of endothelial network formation compared to DMSO, which served as the vehicle. However, cCM from either HLC19-EV and HLC19-PV cells significantly inhibited tubule formation more than the control. Most importantly, cCM from HLC19-PV cells significantly reduced the thickening of the HUVECs network compared to cCM from HLC19-EV cells. In contrast, regorafenib, when coupled with any cCM, slightly reduced tubule formation, regardless of the presence of PRG4 in the cCM; however, its anti-angiogenic effect did not appear to be significantly enhanced by PRG4, suggesting that PRG4 and regorafenib likely influence this process via independent mechanisms (Fig. 6B-D).

### The overexpression of PRG4 by HCC cells shapes their protein secretome towards an overall angiogenesis-restraining effect

To elucidate the mechanisms underlying the reduced HUVEC tubule formation in response to cCM, we profiled angiogenic factors in the conditioned media (cCM) derived from HLC19 PRG4⁺ and PRG4⁻ cells (Fig. 6, E). This revealed the presence of key mediators involved in vessel formation, endothelial cell migration, and extracellular matrix remodeling. Extending the duration of secretome enrichment from 48 to 72 h not only increased the diversity of pro-angiogenic factors but also amplified their abundance. Notably, Activin A, Angiopoietin-1/2, and platelet-derived growth factor (PDGF), which are implicated in endothelial invasion, proliferation, and metastasis, were detected exclusively in the 72-hour secretome. Furthermore, the overall levels of these pro-angiogenic factors were consistently higher in cCM from HLC19 PRG4^−^ cells compared to HLC19 PRG4⁺ cells, supporting a PRG4-mediated suppression of angiogenic signaling (Fig. 6, F). To assess whether differences in the abundance of proteins related to angiogenesis in cCMs from Empty- and PRG4-vector are statistically significant we compared the quantification data related to the two membranes (i.e., PRG4 vector vs. Empty vector, reference category: Empty vector) at 48 and 72 h using the proteins as independent statistical units. At 48 h, the result suggested a trend: *p* = 0.077, PRG4 vector median = 0.354 (interquartile range = IQR = 0.162–0.695), Empty vector median = 0.637 (IQR = 0.441–0.790). At 72 h, a significant difference was observed: *p* = 0.001, PRG4 vector median = 0.061 (IQR = 0.021–0.359), and Empty vector median = 0.217 (IQR = 0.193–0.379).

### PRG4 transcriptomically cooperates with regorafenib to inhibit HCC spheroid expansion

**Fig. 7:**
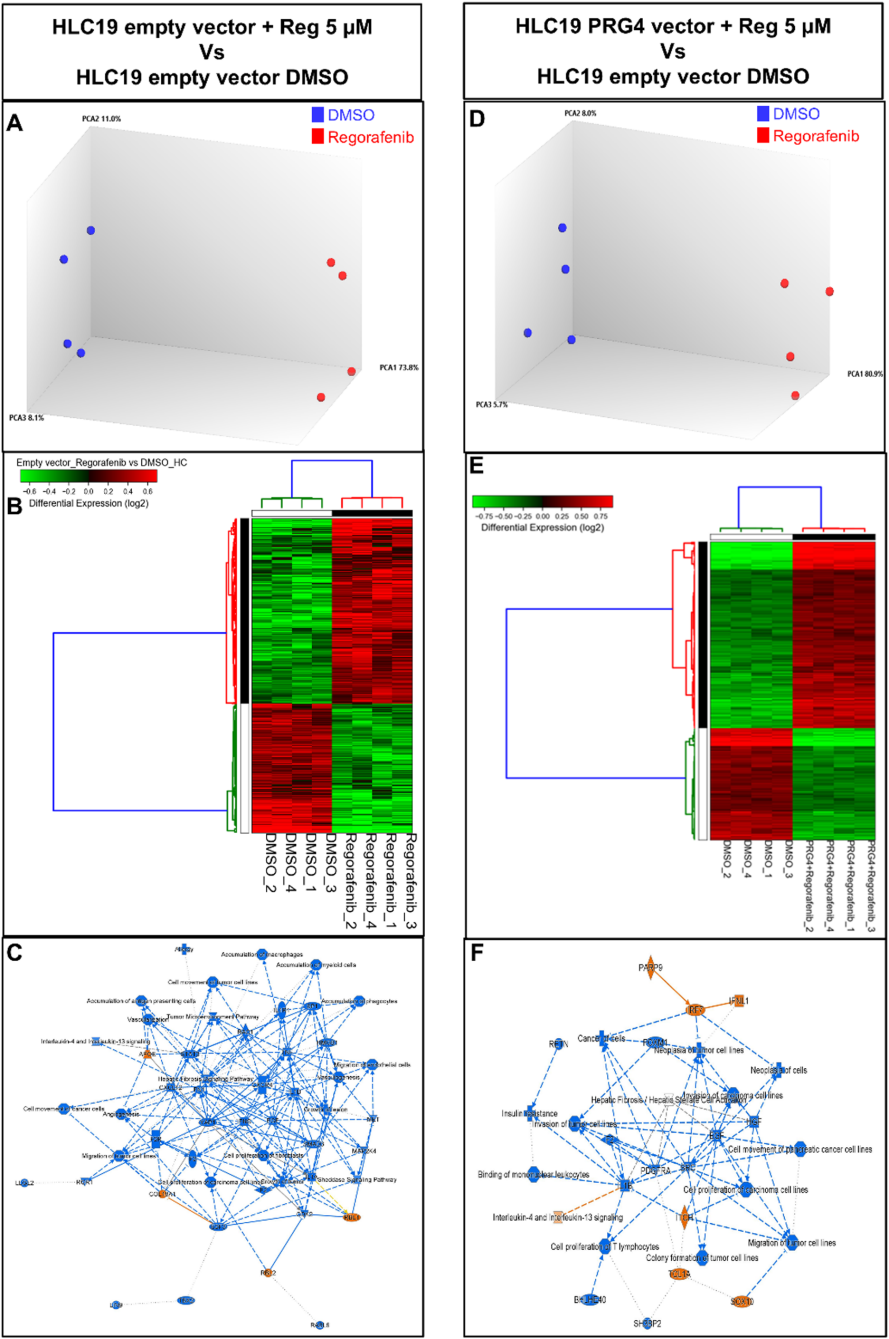
PRG4 expressed within Matrigel-embedded HCC cell tumorspheres cooperates with regorafenib in altering the transcriptomic landscape. Whole transcriptome of cell spheroids made by Empty vector and PRG4 vector HLC19, and treated with DMSO or regorafenib as in 3D growth experiments (Fig. 5) was profiled through Next Generation Sequencing (NGS) analysis. Principal component analysis (PCA, panels **A**, **D**) and hierarchical clustering heatmap using Differently Expressed Genes (DEGs, panel **B**, **E**) shows a clear separation of transcriptomic profiles related to the different treatment conditions. Row represents single genes, while columns represent single samples. The expression levels of genes are coded by the color bar above the heatmap. Gene up-regulation is shown in red whereas down-regulation is shown in green. **C**, **F** From Ingenuity Pathway Analysis (IPA), using the annotations “Disease & Function”, PDGFRA-associated processes result to be down-regulated in regorafenib-treated PRG4^+^ spheroids than untreated PRG4^−^ spheroids, unlike in regorafenib treated PRG4^−^ spheroid versus untreated PRG4^−^ spheroid counterpart, suggesting an impact of PRG4 as an adjuvant on the anti-angiogenic action of regorafenib. Results are from four independent experimental replicates. A further schematic representation of pathways modulated by DEGs is shown in Suppl. Tables 3 and 4

Due to scarcity of xenograft-derived tumor tissues for molecular analyses, a Next Generation Sequencing (NGS) analysis was performed on HLC19 PRG4^−/+^ spheroids treated for 4 days with DMSO vehicle control or regorafenib (Fig. 7A, B, D, E). Differential gene expression and pathway enrichment analysis revealed that regorafenib treatment broadly suppressed several pathways related to cancer cell proliferation, motility, and invasion, as well as angiogenesis, fibrosis, and tumor-promoting immune processes, including IL-1β activity and myeloid cell accumulation (Fig. 7C). It is interestingly to note that combined PRG4 + regorafenib treatment, while inhibiting processes downregulated by regorafenib alone, reduced additional signaling activities, in particular those downstream to platelet-derived growth factor receptor A (PDGFRA) (Fig. 7F). Given PDGFRA’s established role as a promoter of tumor angiogenesis, these findings further corroborate the evidence that PRG4 plays an inhibitory role in neovascularization.

## Discussion

In the present study we support the effectiveness of rhPRG4 in enhancing the anti-proliferative action of regorafenib in a complex HCC in vivo xenograft orthotopic model. Although regorafenib administration has a positive impact on the overall survival of HCC patients, the final benefits resulting from the use of this drug are often overshadowed by reduced patient tolerance. As is the case with several small molecule drugs, regorafenib use may result in unwanted side effects, spanning from mild ailments (such as nausea, diarrhea etc.) to more severe, although less common, toxicity outcomes, including liver and kidney injury. Our results support the proof of principle that using PRG4 as an adjuvant can amplify the anti-proliferative response of regorafenib while maintaining an acceptable tolerance profile for HCC patients regarding this drug’s side effects.

While we had previously demonstrated that PRG4 enhances drug sensitivity in HCC cells through two-dimensional in vitro cell proliferation assays, this study provides more robust support for those findings through a reliable in vivo model [23]. The most commonly employed HCC xenograft models are based on subcutaneous injections of HCC cells, which provide quick tumor development and easy monitoring of mass size evolution. However, in these settings, tumor cells experience a local microenvironment profoundly different from that of a native site wherein hepatic tumors arise and develop. This may lead to a lack of critical stimuli from cells or other liver-specific factors that participate in HCC progression, including the contribution of hepatic stellate cells (HSCs), Kupffer cells, liver sinusoidal endothelial cells, ECM molecules, etc. In contrast, our orthotopic model meets the microenvironmental requirements for the tumorigenic program taking place in human HCC more faithfully.

Although a strong tumor-restricting effect of PRG4 + regorafenib, compared to the drug alone, was observed in our animal model, we are aware of the limitations of this study. The limited knowledge of causative mechanisms bridging PRG4 to its downstream effects undoubtedly warrants further investigation. While we evaluated a synergistic interaction between PRG4 and regorafenib by using the GEE modeling, which captures statistical interactions (population-averaged effects), it is worth to point out that it does not directly quantify pharmacodynamic synergism derived from expected versus observed combination effects, i.e., addressing the biological complexity (mechanism of drug action) and/or pharmacokinetics properties from optimal compound design view.

The lack of synergistic interaction between PRG4 and regorafenib in reducing in vitro endothelial tubulogenesis, as we observed, could be related to the existence of additional levels of complexity behind the function associated with PRG4 activities within the HCC microenvironment.

While the potential use of rhPRG4 in its native form as a cutting-edge adjuvant alongside the standard care of regorafenib is promising, concerns may arise regarding the pharmacodynamics and pharmacokinetics associated with its routes of administration due to the large size of this proteoglycan. A closer insight into the PRG4 structure is therefore essential to identify functional domains required for its anti-tumor activities and whether full-length PRG4 can be tampered with to retain such domains yielding better administrability and bioavailability. A similar approach was pursued to generate endostatin (Endostar™), a C-terminal domain containing a derivative of proteoglycan collagen XVIII, which is produced by cathepsin L-directed proteolytic cleavage. Endostar exhibits anti-angiogenic properties, as it counteracts VEGF, bFGF and FGF-2-mediated vascular tubulogenesis and endothelial cell proliferation [39–41]. We have previously demonstrated that a requisite for PRG4 synergism with regorafenib is its binding to the CD44 HCC cell surface. More recently, researchers have mapped CD44 binding sites within the hemopexin-like C-terminal domain of PRG4 [29, 42]. This prompts us to hypothesize that administering a putative partial PRG4 fragment spanning that region along with regorafenib may result in synergistic anti-proliferative effects that are virtually similar to full-length PRG4.

Besides establishing that CD44 is required for PRG4 to enhance the anti-proliferative activity of regorafenib, we also determined that the expression of CD44 is restricted to HCC cell lines previously characterized as being more aggressive, according to Coulouarn et al. [43]. Before CD44 was discovered to be a PRG4 receptor, its relevance in carcinogenesis was extensively acknowledged, being related to cancer stemness, increased traits of aggressiveness, and resistance to drugs in a spectrum of solid tumors including HCC [44–46]. From a translational perspective, HCC patients who express CD44 on transformed cells may be considered as candidates for PRG4 and regorafenib combinatorial treatment.

## Conclusion

The data presented herein indicate that PRG4 has the potential to enhance the anti-tumor efficacy of regorafenib in the treatment of hepatocellular carcinoma (HCC), without necessitating dose escalation and thereby mitigating the risk of associated side effects. Furthermore, given its endogenous expression across a broad range of human tissues, exogenous administration of PRG4 is anticipated to exhibit a favorable tolerability profile in HCC patients. Collectively, these findings substantiate the rationale for considering PRG4 as a promising adjunct in regorafenib-based therapeutic regimens.

## Supplementary Information

Supplementary Material 1.

Supplementary Material 2.

## Data Availability

The transcriptomic data generated in this study have been deposited in GEO repository and are publicly available under the accession number GSE299840 (https://www.ncbi.nlm.nih.gov/geo/query/acc.cgi?acc=GSE299840).

## Abbreviations

HCC: Hepatocellular carcinoma
PRG4: Proteoglycan-4
2D: Two dimension
3D: Three dimension
G_0_: Gap 0
G_1_: Gap 1
HBV: Hepatitis-B virus
HCV: Hepatitis-C virus
MAFLD: Metabolic-associated fatty liver disease
T2DM: Type 2 diabetes mellitus
CVD: Cardiovascular disease
TACE: Trans-arterial chemoembolization
TKIs: Tyrosine kinase inhibitors
VEGFR: Vascular Endothelial Growth Factor Receptor
CAFs: Cancer-associated fibroblasts
ECM: Extracellular matrix
CD44: Cluster of differentiation-44
CMV: Cytomegalovirus
Reg: Regorafenib
PCNA: Proliferating cell nuclear antigen
VEGFA: Vascular endothelial growth factor A
cCM: concentrated Conditioned Medium
PDGFRA: Platelet Derived Growth Factor Receptor Alpha
bFGF: basic Fibroblast growth factor
FGF2: Fibroblast growth factor
VEGF: Vascular endothelial growth factor
