# Supplementary material for: Proteoglycan-4 potentiates the antitumor efficacy of regorafenib in an orthotopic model of hepatocellular carcinoma
Source: J Exp Clin Cancer Res. 2025 Dec 1;45:7. doi: 10.1186/s13046-025-03575-5 (PMC12771992; doi:10.1186/s13046-025-03575-5)
Supplement: Supplementary file 1 — Supplementary Material 1. [file 13046_2025_3575_MOESM1_ESM.pdf]

Table 1

Antibody	Company/Brand	Cat. no.	Dilution Used
anti-PRG4	Sigma-Aldrich	MABT400	1:5000 – 1:10000
anti-CD44	Cell Signaling	mAb #37259	1:1000
anti-PCNA	Santa Cruz	sc-56	1:1000
anti-VEGFA	Abcam	ab1316	1:1000
anti-ß-actin	Cell Signaling	mAb #3700	1:1000
Goat anti-rabbit IgG, HRP-linked	Cell Signaling	7074S	1:5000
Goat anti-rabbit IgG, HRP-linked	BioRad	1706516	1:5000
anti-ß-tubulin	Cell Signaling	2146S	1:1000
anti-GAPDH (0411) HRP-linked	Santa Cruz	sc-47724	1:1000

Table 2

Gene	Company/Brand	Assay Type	Primer ID
PRG4	BioRad	SYBR® Green	qHsaCED0043693
CD44	BioRad	SYBR® Green	qHsaCID0013679
VEGFA	BioRad	SYBR® Green	qHsaCED0006937
ACTB	BioRad	SYBR® Green	qHsaCED0036269

Suppl. Tables 1 and 2. Antibodies and primers used.

Suppl. Table 3: Summary of pathways impacted by DEGs comparing the effect of Regorafenib treatment in HLC19-empty vector versus DMSO (vehicle) in HLC19-empty vector. Pathways generated by Ingenuity Pathway Analysis (IPA) by Qiagen are sorted by -log(p-value) with a cut-off of ≥2 (p ≤ 0.01), where z-score determines the direction of pathway activation/inhibition.

Ingenuity Canonical Pathways	-log(p-value)	z-score
Granzyme A Signaling	4E-01	1
Tumor Microenvironment Pathway	1.05E+01	-3.157
Interleukin-4 and Interleukin-13 signaling	1.04E+01	-1.225
Sheddase Signaling Pathway	9.09E+00	-1.257
Role of Tissue Factor in Cancer	8.88E+00	-2.556
Wound Healing Signaling Pathway	8.65E+00	-1.061
Hepatic Fibrosis Signaling Pathway	8.39E+00	-3.042
Extracellular matrix organization	8.38E+00	0.655
HMGB1 Signaling	7.71E+00	-1.886
Assembly of collagen fibrils and other multimeric structures	7.55E+00	1.604
Interleukin-10 signaling	7.53E+00	-3.051
Activin Inhibin Signaling Pathway	7.50E+00	-2.043
HGF Signaling	7.44E+00	-2.138
Integrin cell surface interactions	7.03E+00	-0.243
IL-8 Signaling	6.56E+00	-2.711
Acute Phase Response Signaling	6.19E+00	-2.668
HIF1α Signaling	5.94E+00	-2.858
IL-17A Signaling in Fibroblasts	5.90E+00	-2.496
Regulation of Insulin-like Growth Factor (IGF) transport and uptake by IGFBPs	5.89E+00	-2.065
Post-translational protein phosphorylation	5.55E+00	-2.183
RAF-independent MAPK1/3 activation	5.53E+00	-2.121
Response to elevated platelet cytosolic Ca2+	5.47E+00	1.147
Role of JAK family kinases in IL-6-type Cytokine Signaling	5.12E+00	-2.309
Cholecystokinin/Gastrin-mediated Signaling	5.02E+00	-1.807
Erythropoietin Signaling Pathway	5.00E+00	-0.426
Coagulation System	4.93E+00	-0.707
Molecular Mechanisms of Cancer	4.91E+00	-1.016
GDNF Family Ligand-Receptor Interactions	4.74E+00	-0.302
IL-17 Signaling	4.70E+00	-1.964
Platelet Adhesion to exposed collagen	4.48E+00	-0.816
Degradation of the extracellular matrix	4.44E+00	-0.577
GADD45 Signaling	4.39E+00	2.53
Apelin Endothelial Signaling Pathway	4.33E+00	-1.732
RAR Activation	4.28E+00	1
Differential Regulation of Cytokine Production in Macrophages and T Helper Cells by IL-17A and IL-17F	4.15E+00	-2.236
Collagen degradation	4.12E+00	0.905
Signaling by Rho Family GTPases	4.08E+00	-0.728
Elastic fibre formation	4.08E+00	-0.333
Collagen chain trimerization	4.08E+00	1.667
Macropinocytosis Signaling	4.07E+00	-1.667
Nucleotide catabolism	4.05E+00	0.707
Syndecan interactions	3.98E+00	-1.134

ILK Signaling	3.96E+00	-1.414
Collagen biosynthesis and modifying enzymes	3.94E+00	1.508
TREM1 Signaling	3.91E+00	-3.317
Hematoma Resolution Signaling Pathway	3.89E+00	1.225
Dissolution of Fibrin Clot	3.87E+00	-1.342
PI3K/AKT Signaling	3.86E+00	-2.53
RAF/MAP kinase cascade	3.81E+00	-1.043
ERK/MAPK Signaling	3.71E+00	-1
Protein Kinase A Signaling	3.68E+00	1.512
Macrophage Alternative Activation Signaling Pathway	3.66E+00	0.229
NOD1/2 Signaling Pathway	3.63E+00	-2.065
NAFLD Signaling Pathway	3.60E+00	-2.558
Regulation of the Epithelial Mesenchymal Transition by Growth Factors Pathway	3.60E+00	-1.698
RAC Signaling	3.60E+00	-1.667
LPS-stimulated MAPK Signaling	3.60E+00	-1.508
L1CAM interactions	3.58E+00	-0.258
CXCR4 Signaling	3.50E+00	-1.387
IL-10 Signaling	3.46E+00	2
Thrombopoietin Signaling	3.45E+00	-2.333
Human Embryonic Stem Cell Pluripotency	3.43E+00	-2.524
Aldosterone Signaling in Epithelial Cells	3.41E+00	-0.707
Regulation of Cellular Mechanics by Calpain Protease	3.41E+00	0.447
IL-6 Signaling	3.39E+00	-2.138
Signaling by NOTCH1	3.39E+00	0.905
Kinetochore Metaphase Signaling Pathway	3.36E+00	-0.707
LXR/RXR Activation	3.36E+00	3.207
RHO GTPase cycle	3.30E+00	-1.521
Docosahexaenoic Acid (DHA) Signaling	3.30E+00	0.655
IL-7 Signaling Pathway	3.29E+00	-1.265
CD40 Signaling	3.29E+00	-0.333
Signaling by MET	3.29E+00	-0.302
Cell junction organization	3.28E+00	1.508
Paxillin Signaling	3.24E+00	0.378
NRF2-mediated Oxidative Stress Response	3.23E+00	-1.941
Phagosome Formation	3.22E+00	-1.155
Signaling by VEGF	3.20E+00	-2.496
S100 Family Signaling Pathway	3.19E+00	-1.697
Endothelin-1 Signaling	3.18E+00	-0.728
IL-15 Production	3.15E+00	-1.069
FXR/RXR Activation	3.15E+00	2.357
G-Protein Coupled Receptor Signaling	3.12E+00	-1.443
Neutrophil Extracellular Trap Signaling Pathway	3.11E+00	-1.826
Cell surface interactions at the vascular wall	3.05E+00	-2.236
Cyclins and Cell Cycle Regulation	2.98E+00	-2.121
Signaling by ERBB2	2.94E+00	-2.828
PDGF Signaling	2.94E+00	-1.897
NGF-stimulated transcription	2.93E+00	-2.646
Interconversion of nucleotide di- and triphosphates	2.92E+00	-0.816
DHCR24 Signaling Pathway	2.92E+00	0.775

Reversible hydration of carbon dioxide	2.91E+00	-2
Ribonucleotide Reductase Signaling Pathway	2.89E+00	-0.5
Nuclear Cytoskeleton Signaling Pathway	2.88E+00	-1.789
Mitotic G1 phase and G1/S transition	2.81E+00	1.941
Mitotic Prophase	2.80E+00	-3.464
PAK Signaling	2.80E+00	-0.378
RET signaling	2.80E+00	-0.378
Actin Nucleation by ARP-WASP Complex	2.77E+00	-1
P2Y Purinergic Receptor Signaling Pathway	2.75E+00	-0.905
Role of Hypercytokinemia/hyperchemokinemia in the Pathogenesis of Influenza	2.74E+00	-1.667
UVB-Induced MAPK Signaling	2.71E+00	-1.414
ERBB Signaling	2.70E+00	-1.265
Mitotic Roles of Polo-Like Kinase	2.68E+00	-1.342
Telomerase Signaling	2.66E+00	-1.414
CGAS-STING Signaling Pathway	2.63E+00	-2.496
MAPK targets/ Nuclear events mediated by MAP kinases	2.61E+00	-1.633
Gαq Signaling	2.61E+00	-1.604
Neutrophil degranulation	2.59E+00	-0.686
Cell Cycle: G1/S Checkpoint Regulation	2.59E+00	1.414
TGF-β Signaling	2.55E+00	-1.414
Relaxin Signaling	2.55E+00	-0.378
Signaling by NOTCH2	2.54E+00	1.633
D-myo-inositol-5-phosphate Metabolism	2.53E+00	-1.291
Mitotic Prometaphase	2.51E+00	-4.243
MSP-RON Signaling in Cancer Cells Pathway	2.49E+00	-1.604
FGF Signaling	2.45E+00	-0.632
Production of Nitric Oxide and Reactive Oxygen Species in Macrophages	2.44E+00	-1
Superpathway of Inositol Phosphate Compounds	2.44E+00	-0.943
Hepatic Cholestasis	2.43E+00	-2.524
Semaphorin Neuronal Repulsive Signaling Pathway	2.38E+00	1.155
IL-33 Signaling Pathway	2.37E+00	-2.84
ABRA Signaling Pathway	2.37E+00	-1.897
IL-17A Signaling in Gastric Cells	2.36E+00	-1.342
Nucleosome assembly	2.35E+00	-2.646
PFKFB4 Signaling Pathway	2.35E+00	-0.816
Plasma lipoprotein assembly, remodeling, and clearance	2.31E+00	-0.333
Signaling by NOTCH3	2.30E+00	1.89
PPARβγ/RXRβ Activation	2.30E+00	2.673
IGF-1 Signaling	2.29E+00	-1.89
Effects of PIP2 hydrolysis	2.29E+00	-1.342
3-phosphoinositide Biosynthesis	2.29E+00	-1.291
Cell Cycle Regulation by BTG Family Proteins	2.29E+00	-0.447
Fc Epsilon RI Signaling	2.28E+00	-0.905
PTEN Signaling	2.28E+00	2.828
Toll-like Receptor Signaling	2.27E+00	-1.89
3-phosphoinositide Degradation	2.26E+00	-1.291
UVC-Induced MAPK Signaling	2.25E+00	-1.633
ERB2-ERBB3 Signaling	2.25E+00	-1.633
Transcriptional regulation of granulopoiesis	2.25E+00	-0.378

Formation of Fibrin Clot (Clotting Cascade)	2.24E+00	-0.816
VDR/RXR Activation	2.23E+00	-0.378
ID1 Signaling Pathway	2.22E+00	-1.5
Ephrin Receptor Signaling	2.22E+00	-1.414
Caveolar-mediated Endocytosis Signaling	2.19E+00	-1.414
RHOGDI Signaling	2.19E+00	0.707
PPAR Signaling	2.19E+00	3.162
Cell Cycle Checkpoints	2.18E+00	-4.146
Hepatitis B Chronic Liver Pathogenesis Signaling Pathway	2.16E+00	-1.807
Beta-catenin independent WNT signaling	2.16E+00	-0.258
ISGylation Signaling Pathway	2.16E+00	0.302
G alpha (12/13) signalling events	2.16E+00	1
D-myo-inositol (1,4,5,6)-Tetrakisphosphate Biosynthesis	2.14E+00	-1.387
D-myo-inositol (3,4,5,6)-tetrakisphosphate Biosynthesis	2.14E+00	-1.387
Thrombin Signaling	2.13E+00	-0.632
IL-3 Signaling	2.12E+00	-2.121
Chemokine Signaling	2.12E+00	-1.89
Transcriptional regulation by RUNX3	2.11E+00	-1.342
Actin Cytoskeleton Signaling	2.10E+00	-1.265
IL-12 Signaling and Production in Macrophages	2.10E+00	-0.688
Integrin Signaling	2.06E+00	-1.069
IL-1 Signaling	2.05E+00	-1.134
Interferon gamma signaling	2.05E+00	1.265
Leukocyte Extravasation Signaling	2.04E+00	-1.387
PIP3 activates AKT signaling	2.03E+00	-2.496
EGF Signaling	2.03E+00	-1.89
Transport of vitamins, nucleosides, and related molecules	2.03E+00	0.816
Transport of bile salts and organic acids, metal ions and amine compounds	2.02E+00	-1
Eicosanoid Signaling	2.01E+00	-0.218

Table 4: Summary of pathways impacted by DEGs comparing the effect of Regorafenib treatment in HLC19-PRG4 vector versus DMSO (vehicle) in HLC19-PRG4 vector. Pathways generated by Ingenuity Pathway Analysis (IPA) Qiagen are sorted by -log(p-value) with a cut-off of ≥2 (p ≤ 0.01), where z-score determines the direction of pathway activation/inhibition.

HLC19 PRG4 vector + Reg 5 µM versus HLC19 empty vector DMSO

**Ingenuity Canonical Pathways**	**-log(p-value)**	**z-score**
Wound Healing Signaling Pathway	1.51E+01	0.885
Interleukin-4 and Interleukin-13 signaling	1.37E+01	0.73
Tumor Microenvironment Pathway	1.27E+01	-0.507
RAR Activation	1.19E+01	1,987
Pathogen Induced Cytokine Storm Signaling Pathway	1.16E+01	-0.566
Collagen biosynthesis and modifying enzymes	9.33E+00	2,065
Activin Inhibin Signaling Pathway	9.24E+00	-1,183
Assembly of collagen fibrils and other multimeric structures	9.18E+00	1,414
Sheddase Signaling Pathway	9.11E+00	-1,372
Role of Tissue Factor in Cancer	8.88E+00	-1,219
HMGB1 Signaling	8.73E+00	-1,342
Macrophage Classical Activation Signaling Pathway	8.25E+00	-0.392
Hepatic Cholestasis	7.98E+00	-1,414
Molecular Mechanisms of Cancer	7.92E+00	1,013
Extracellular matrix organization	7.82E+00	1,706
IL-17 Signaling	7.62E+00	-0.928
Interferon alpha/beta signaling	7.52E+00	2,357
Erythropoietin Signaling Pathway	7.48E+00	1,177
HIF1Î± Signaling	7.10E+00	-0.73
Neuroinflammation Signaling Pathway	6.99E+00	0.539
NAFLD Signaling Pathway	6.97E+00	-2,121
NOD1/2 Signaling Pathway	6.85E+00	-2,117
Regulation of the Epithelial Mesenchymal Transition by Growth Factors Pathway	6.81E+00	0.408
S100 Family Signaling Pathway	6.81E+00	0.843
Collagen chain trimerization	6.81E+00	1,941
Neutrophil degranulation	6.79E+00	1,941
IL-10 Signaling	6.60E+00	0.209
Eicosanoid Signaling	6.51E+00	1,219
Neutrophil Extracellular Trap Signaling Pathway	6.47E+00	-1,677
GP6 Signaling Pathway	6.37E+00	1,279
STAT3 Pathway	6.32E+00	-0.243
Role of Macrophages, Fibroblasts and Endothelial Cells in Rheumatoid Arthritis	6.31E+00	-0.973
IL-6 Signaling	6.31E+00	-0.229
Collagen degradation	6.31E+00	1,807
Hepatic Fibrosis Signaling Pathway	6.26E+00	-0.949
IL-8 Signaling	6.16E+00	-0.392
DHCR24 Signaling Pathway	6.10E+00	2,400
RHO GTPase cycle	6.05E+00	-0.866
Acute Phase Response Signaling	5.90E+00	-0.243
Macrophage Alternative Activation Signaling Pathway	5.85E+00	1,800

FXR/RXR Activation	5.72E+00	2,041
IL-17A Signaling in Fibroblasts	5.69E+00	-1,069
LXR/RXR Activation	5.66E+00	1,886
Apelin Endothelial Signaling Pathway	5.43E+00	-0.258
Syndecan interactions	5.38E+00	1,000
Integrin cell surface interactions	5.33E+00	2,000
Protein Kinase A Signaling	5.31E+00	1,372
Coagulation System	5.23E+00	-1,667
VDR/RXR Activation	5.16E+00	0.302
Interferon gamma signaling	5.12E+00	3,153
MSP-RON Signaling in Macrophages Pathway	5.05E+00	0.728
Interleukin-10 signaling	4.97E+00	-2,714
Role of JAK family kinases in IL-6-type Cytokine Signaling	4.95E+00	-1,387
CGAS-STING Signaling Pathway	4.70E+00	-1,606
Hepatitis B Chronic Liver Pathogenesis Signaling Pathway	4.56E+00	0.408
ID1 Signaling Pathway	4.49E+00	1,225
IL-12 Signaling and Production in Macrophages	4.41E+00	-0.756
Relaxin Signaling	4.41E+00	1,155
GNRH Signaling	4.37E+00	0.471
Endothelin-1 Signaling	4.33E+00	0.853
Degradation of the extracellular matrix	4.31E+00	0.535
O-linked glycosylation	4.29E+00	0.243
Elastic fibre formation	4.28E+00	1,897
Potassium Channels	4.25E+00	3,000
G-Protein Coupled Receptor Signaling	4.11E+00	0.391
PPAR Signaling	3.99E+00	1,604
HGF Signaling	3.98E+00	-0.832
RHO GTPases Activate NADPH Oxidases	3.88E+00	0.378
IL-33 Signaling Pathway	3.86E+00	-1,342
P2Y Purinergic Receptor Signaling Pathway	3.85E+00	0.775
Aryl Hydrocarbon Receptor Signaling	3.84E+00	-1,698
Regulation of Insulin-like Growth Factor (IGF) transport and uptake by IGFBPs	3.78E+00	0.243
Differential Regulation of Cytokine Production in Macrophages and T Helper Cells by IL-17A and IL-17F	3.75E+00	-2,236
Docosahexaenoic Acid (DHA) Signaling	3.74E+00	1,633
HEY1 Signaling Pathway	3.74E+00	2,524
Ribonucleotide Reductase Signaling Pathway	3.72E+00	-0.447
MyD88:MAL(TIRAP) cascade initiated on plasma membrane	3.70E+00	1,000
ERBB Signaling	3.64E+00	0.277
Th2 Pathway	3.57E+00	-0.258
Dissolution of Fibrin Clot	3.53E+00	-1,342
MSP-RON Signaling in Cancer Cells Pathway	3.50E+00	-0.728
Leukocyte Extravasation Signaling	3.48E+00	1,414
Apelin Liver Signaling Pathway	3.47E+00	1,633
Response to elevated platelet cytosolic Ca2+	3.45E+00	1,698
Cohesin Chromatin Regulation Pathway	3.42E+00	1,000
Cardiac Î^2-adrenergic Signaling	3.42E+00	1,941
IL-1 Signaling	3.40E+00	-0.333
IL-27 Signaling Pathway	3.38E+00	0.728

Interferon Signaling	3.37E+00	1,633
CD40 Signaling	3.31E+00	1,000
Th1 Pathway	3.25E+00	1,604
CLEAR Signaling Pathway	3.21E+00	0.756
Cell junction organization	3.16E+00	-0.277
Differential Regulation of Cytokine Production in Intestinal Epithelial Cells by IL-17A and IL-17F	3.11E+00	-2,236
Cholecystokinin/Gastrin-mediated Signaling	3.09E+00	-0.535
Platelet Adhesion to exposed collagen	3.05E+00	-0.447
UVC-Induced MAPK Signaling	3.04E+00	-0.707
Thrombin Signaling	3.03E+00	1,500
Interleukin-6 family signaling	3.00E+00	-1,633
Fc Epsilon RI Signaling	2.98E+00	0.577
PPARÎ±/RXRÎ± Activation	2.97E+00	1,000
Inhibition of Angiogenesis by TSP1	2.96E+00	-1,342
fMLP Signaling in Neutrophils	2.96E+00	0.832
HER-2 Signaling in Breast Cancer	2.92E+00	-2,294
Superpathway of Inositol Phosphate Compounds	2.92E+00	1,342
Oncostatin M Signaling	2.91E+00	-0.816
IL-15 Production	2.91E+00	-0.258
Renin-Angiotensin Signaling	2.91E+00	0.302
Antigen Presentation Pathway	2.91E+00	1,633
PDGF Signaling	2.86E+00	-0.905
UVB-Induced MAPK Signaling	2.85E+00	-0.333
Macropinocytosis Signaling	2.84E+00	0.378
Plasma lipoprotein assembly, remodeling, and clearance	2.84E+00	0.905
D-myo-inositol-5-phosphate Metabolism	2.80E+00	1,213
Glycine Betaine Degradation	2.80E+00	2,000
Toll-like Receptor Signaling	2.79E+00	-1,667
Phagosome Formation	2.79E+00	1,109
Phase I - Functionalization of compounds	2.78E+00	3,742
RAF/MAP kinase cascade	2.76E+00	-0.626
MIF Regulation of Innate Immunity	2.72E+00	-0.378
G alpha (12/13) signalling events	2.66E+00	1,508
Glutaminergic Receptor Signaling Pathway (Enhanced)	2.64E+00	1,512
Cell Cycle: G1/S Checkpoint Regulation	2.64E+00	1,667
Signaling by PDGF	2.63E+00	0.333
IL-3 Signaling	2.61E+00	-0.333
Neuregulin Signaling	2.60E+00	-0.333
PAK Signaling	2.60E+00	1,508
Inhibition of Matrix Metalloproteases	2.59E+00	-1,342
Epithelial Adherens Junction Signaling	2.58E+00	-1,698
CDK5 Signaling	2.58E+00	-0.333
cAMP-mediated signaling	2.58E+00	2,837
Sphingosine-1-phosphate Signaling	2.57E+00	0.905
Actin Cytoskeleton Signaling	2.56E+00	1,508
Orexin Signaling Pathway	2.56E+00	2,837
PI3K/AKT Signaling	2.55E+00	-2,121
3-phosphoinositide Degradation	2.55E+00	1,000
iNOS Signaling	2.54E+00	-1,134

NGF-stimulated transcription	2.52E+00	-1,134
Telomerase Signaling	2.51E+00	-0.333
Platelet Aggregation (Plug Formation)	2.50E+00	2,000
eNOS Signaling	2.49E+00	1,387
Natural Killer Cell Signaling	2.48E+00	-0.688
Signaling by NOTCH3	2.48E+00	2,121
Crosstalk between Dendritic Cells and Natural Killer Cells	2.42E+00	0.333
Transcriptional regulation of granulopoiesis	2.42E+00	0.707
UVA-Induced MAPK Signaling	2.42E+00	1,890
FGF Signaling	2.41E+00	0.302
Cyclophilin Signaling Pathway	2.41E+00	1,460
Production of Nitric Oxide and Reactive Oxygen Species in Macrophages	2.40E+00	0.229
Cellular hexose transport	2.38E+00	-0.447
Regulation of TLR by endogenous ligand	2.38E+00	1,342
HOTAIR Regulatory Pathway	2.38E+00	1,500
LPS/IL-1 Mediated Inhibition of RXR Function	2.31E+00	-0.905
RAF-independent MAPK1/3 activation	2.29E+00	-1,342
Apelin Cardiac Fibroblast Signaling Pathway	2.29E+00	0.447
Gαq Signaling	2.28E+00	1,000
Transport of vitamins, nucleosides, and related molecules	2.27E+00	1,134
Acetylcholine Receptor Signaling Pathway	2.26E+00	0.688
Mouse Embryonic Stem Cell Pluripotency	2.25E+00	0.302
Activation of Matrix Metalloproteinases	2.25E+00	0.816
Synthesis of Prostaglandins (PG) and Thromboxanes (TX)	2.25E+00	1,000
G Beta Gamma Signaling	2.22E+00	0.277
Cell surface interactions at the vascular wall	2.22E+00	0.894
Retinoid metabolism and transport	2.22E+00	2,646
Signaling by MET	2.20E+00	1,265
SPINK1 General Cancer Pathway	2.20E+00	1,414
PIP3 activates AKT signaling	2.18E+00	-0.258
IGF-1 Signaling	2.18E+00	0.333
Ephrin Receptor Signaling	2.17E+00	-0.302
Arachidonic acid metabolism	2.16E+00	1,890
tRNA Splicing	2.16E+00	1,890
Apoptosis Signaling	2.15E+00	1,667
Chemokine Signaling	2.13E+00	-0.707
Netrin Signaling	2.13E+00	0.243
GPVI-mediated activation cascade	2.13E+00	0.816
Nucleotide catabolism	2.13E+00	1,633
Gαi Signaling	2.13E+00	2,111
Xenobiotic Metabolism CAR Signaling Pathway	2.13E+00	2,324
Immunogenic Cell Death Signaling Pathway	2.08E+00	-0.905
Xenobiotic Metabolism General Signaling Pathway	2.08E+00	0.577
Integration of energy metabolism	2.08E+00	1,155
CCR3 Signaling in Eosinophils	2.05E+00	1,000
WNT/SHH Axonal Guidance Signaling Pathway	2.03E+00	0.258
TGF-β Signaling	2.02E+00	-1,000
Detoxification of Reactive Oxygen Species	2.01E+00	-0.816
p38 MAPK Signaling	2.01E+00	0.632

Suppl. Fig. 1

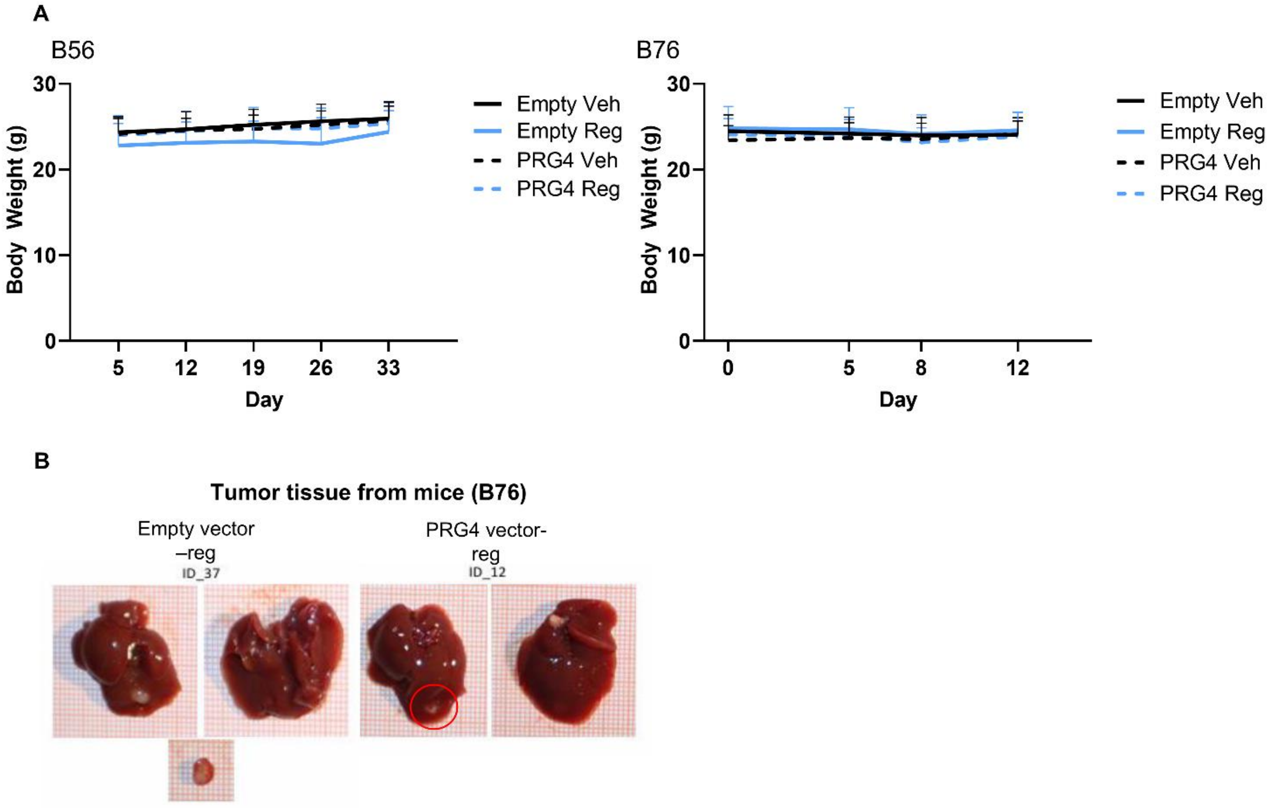

Suppl. Fig. 1. (**A**) Body weight graphs of mice to assess animal welfare in in-vivo studies B56 (***left***) and B76 (***right***). (**B**) Images of livers bearing tumors explanted for analysis by western blot of protein expression shown in fig. 2F.

Suppl. Fig. 2

LIVER HEPATOCELLULAR CARCINOMA (TCGA) - Survival analysis

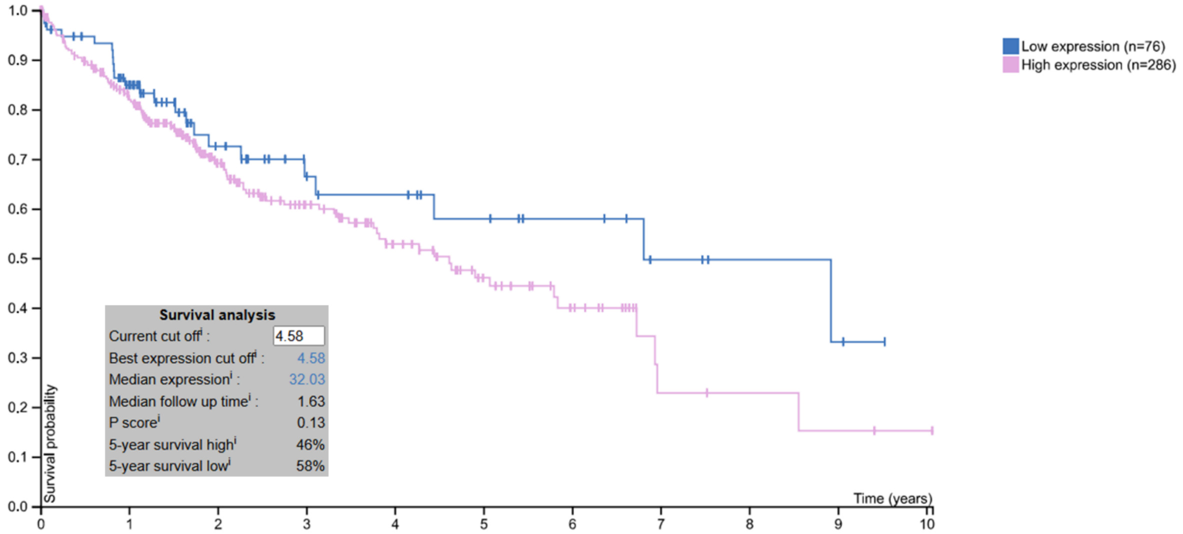

LIVER HEPATOCELLULAR CARCINOMA (validation) - Survival analysis

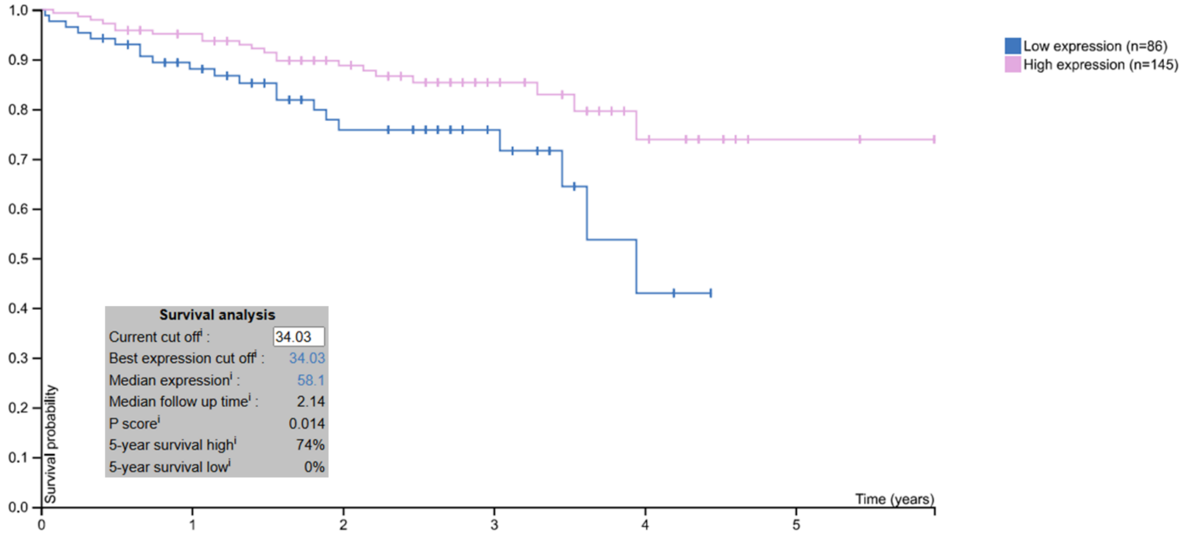

Suppl. Fig. 2. Survival curves (Kaplan-Meier) of patients with HCC based on high and low tissue expression of PRG4 mRNA relative to the median value. Data sourced from "The Human Protein Atlas" (https://www.proteinatlas.org/ENSG00000116690-PRG4/cancer/liver+cancer).

Suppl. Fig. 3

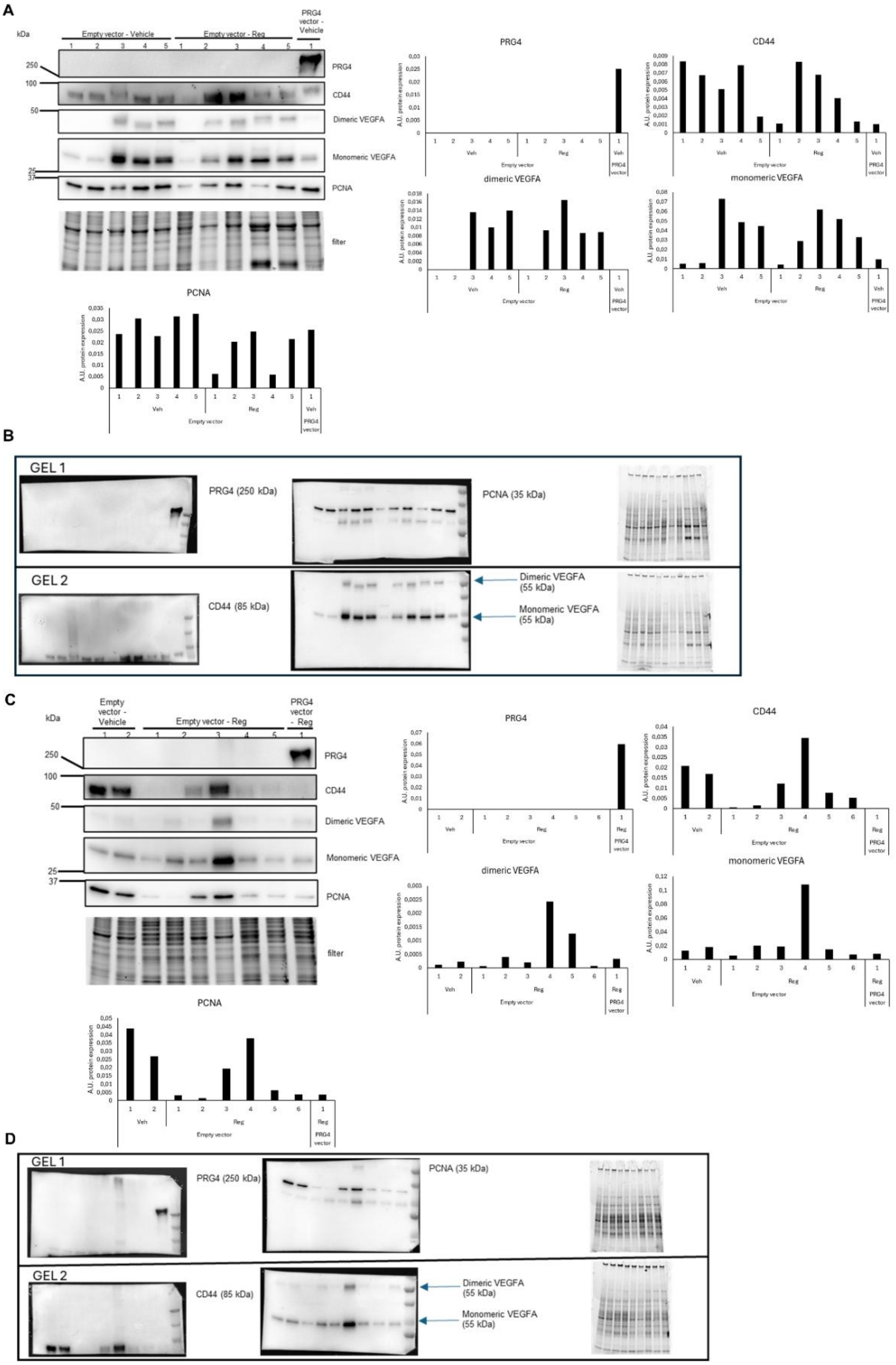

Suppl. Fig. 3. Western blots on tumor samples from in vivo intrahepatic HCC experimental models (studies B56 and B76). (**A**) Blots of tumor samples from B56 study and densitometry quantification; (**B**) Full blots relating to the panel A. (**C**) Blots of tumor samples from B76 study and densitometry quantification. (**D**) Full blots relating to the panel C. Densitometry data are normalized for total protein signal through Tris-Glycine eXtended (TGX) fluorescence detection (filter). A.U.: arbitrary units.

Suppl. Fig. 4

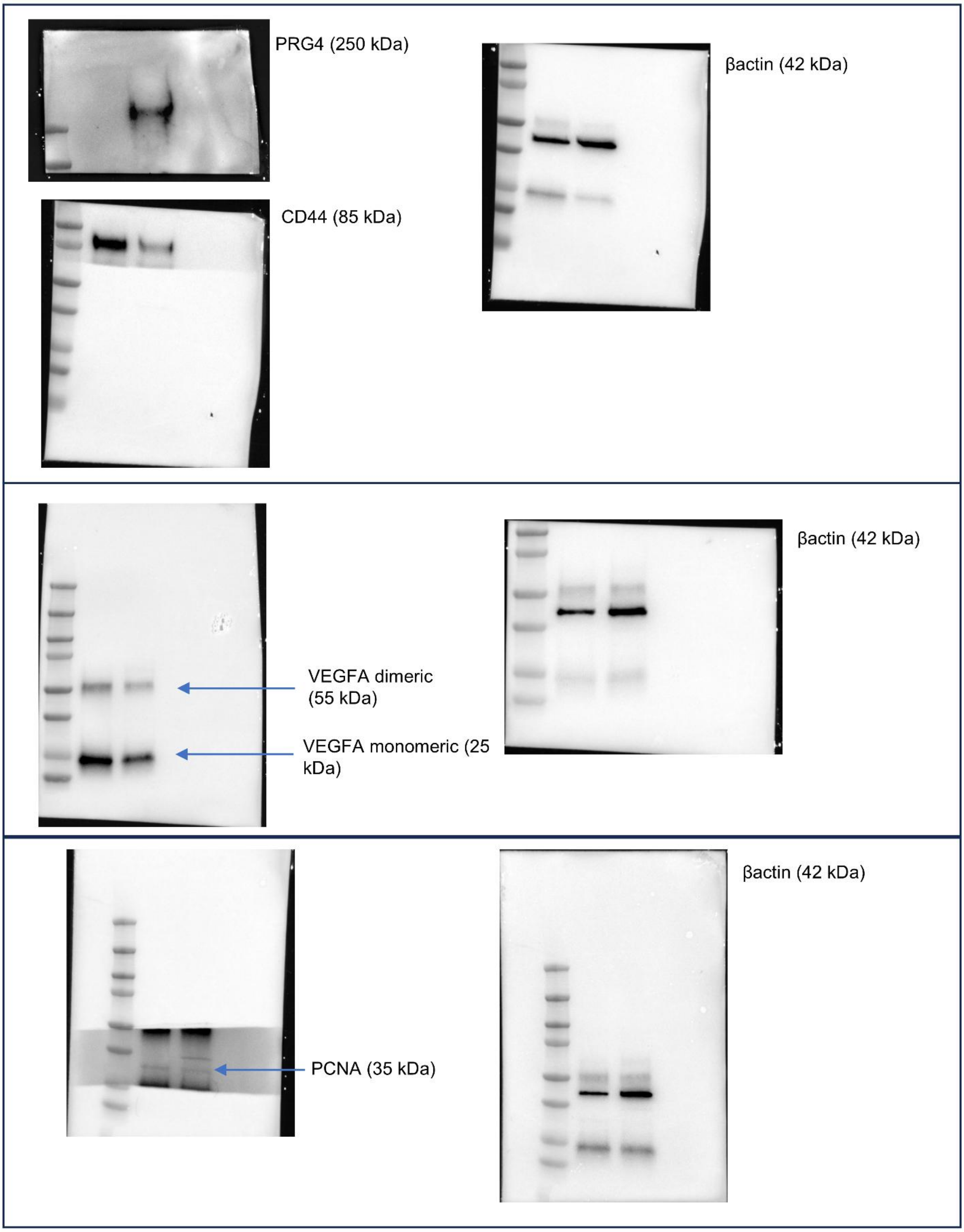

Suppl. Fig. 4. Full western blot images for Figure 2F.

Suppl. Fig. 5

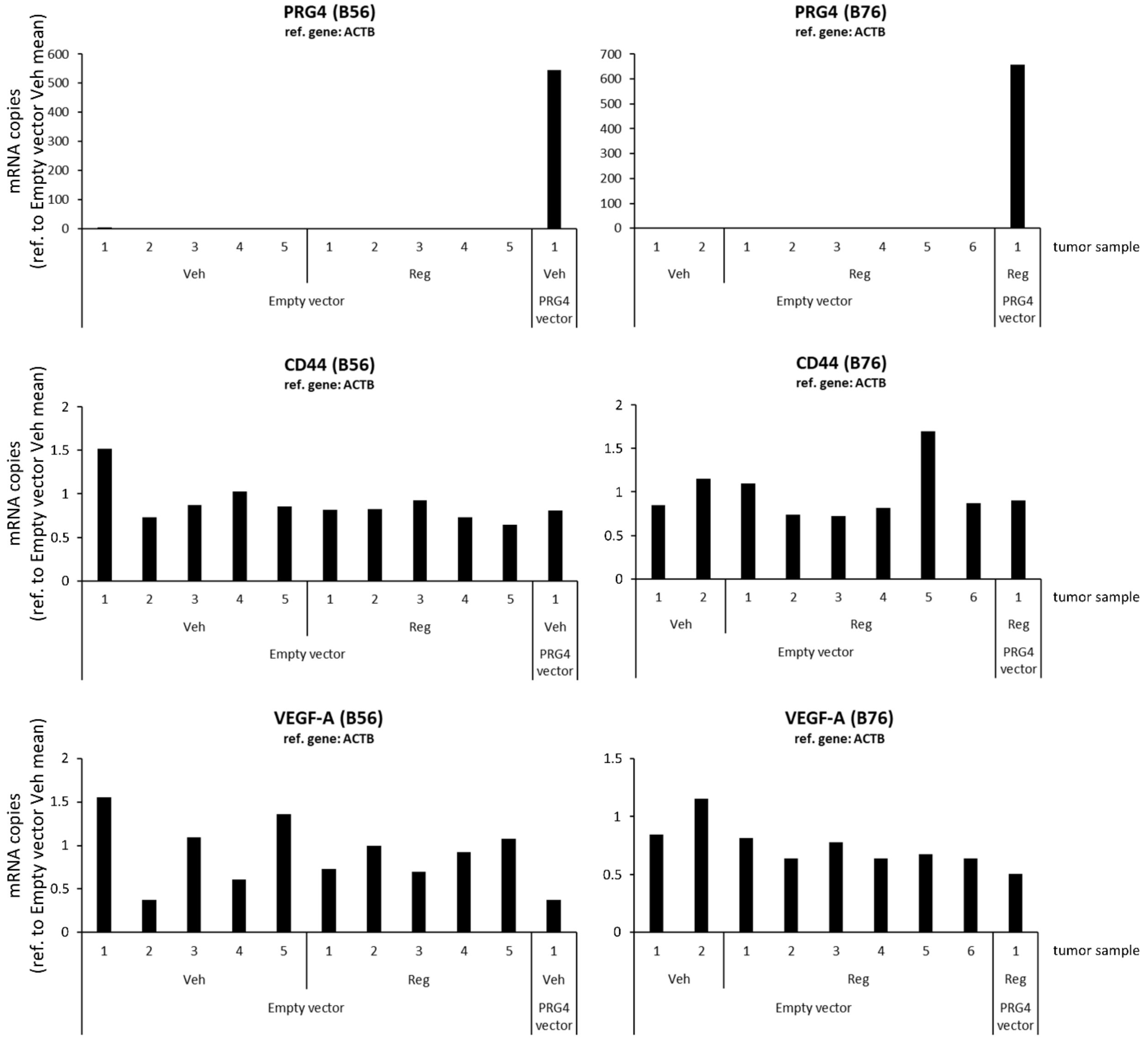

Suppl. Fig. 5. Quantitative PCR (qPCR) on tumor specimens collected at the endpoint of *in-vivo* HCC experiments (B56, B76)

Suppl. Fig. 6

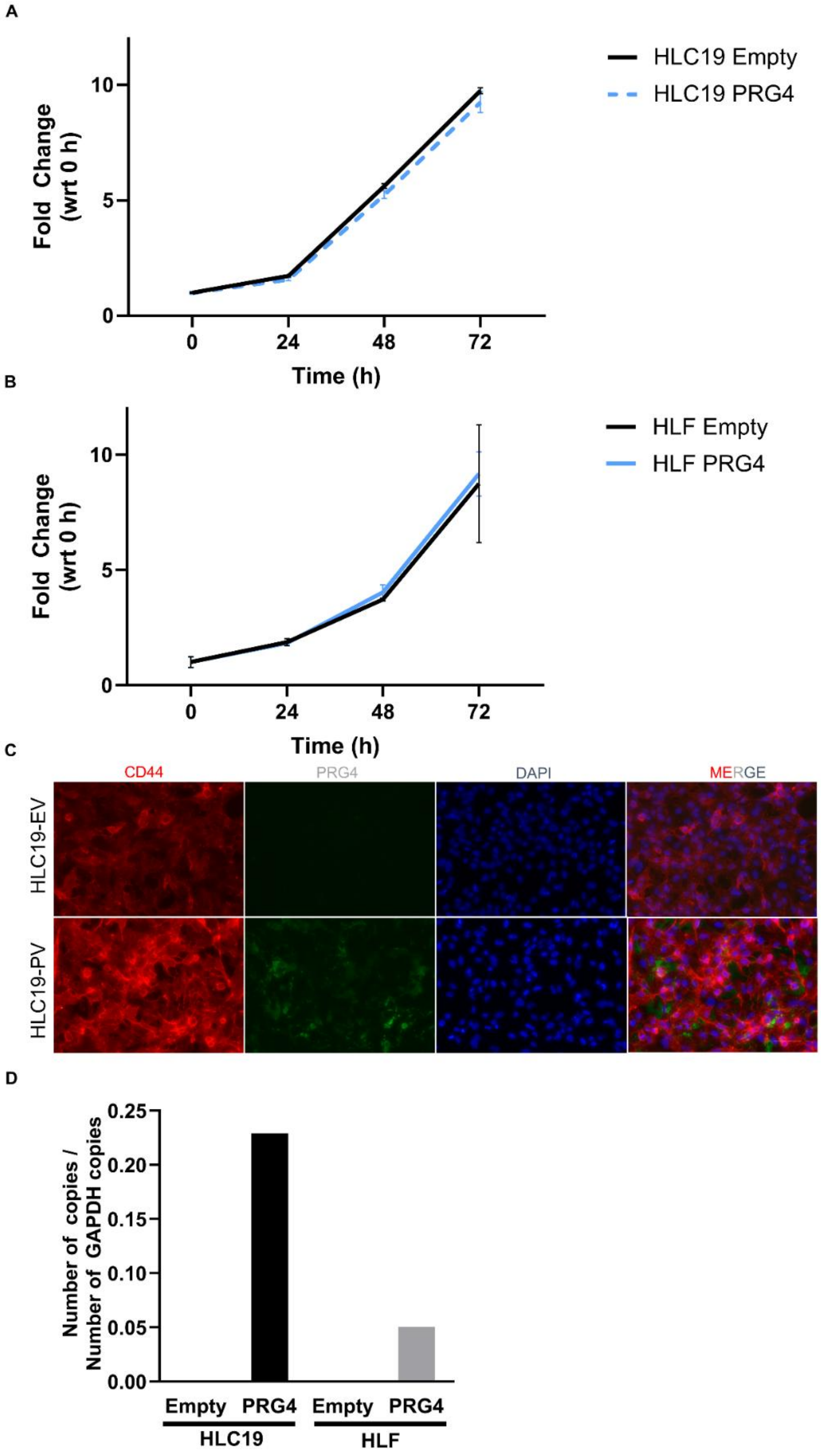

Suppl. Fig. 6. (**A**) Proliferation curve of empty vector and PRG4 vector HLC19 cells. Black solid line: HLC19 empty vector cell line; blue dashed line: HLC19 PRG4 vector. (**B**) Proliferation curve of empty vector and PRG4 vector HLF cells. Black solid line: HLF empty vector cell line; blue dashed line: HLF PRG4 vector. (**C**) 2D immunofluorescence staining of HLC19 empty vector and PRG4 vector: The cells were stained for CD44 (red), PRG4 (green) anche nuclei were counterstained with DAPI (blue). Merged images show co-localization of marker. Scale bar: 100 µm. (**D**) Quantification of number of copies of PRG4 in HLC19 and HLF cell line transfected with empty vector and PRG4 vector.

Suppl. Fig. 7

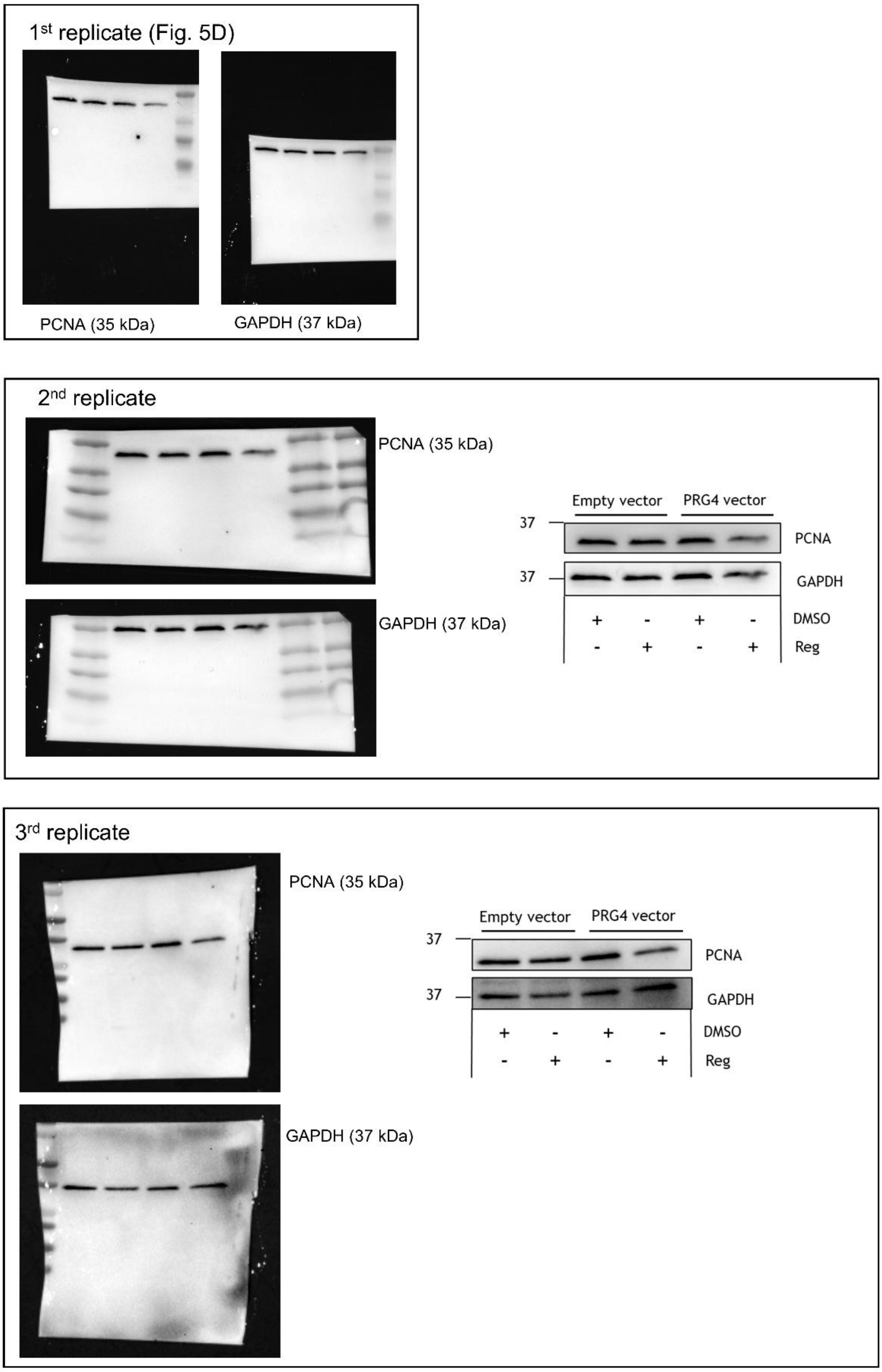

Suppl. Fig. 7. (**A**) Full western blot images for the 1st replicate of figure 5D in the main article. The blots show PCNA and GAPDH exposure and ladder as reference. (**B, C, *left***) Full western blot of the second and third replicate used for the quantification of PCNA expression in the statistical analysis; (**B,C, *right***) complete panel showing all samples and corresponding treatments for protein expression analysis.
