# Supplementary material for: Proteoglycan-4 potentiates the antitumor efficacy of regorafenib in an orthotopic model of hepatocellular carcinoma
Source: J Exp Clin Cancer Res. 2025 Dec 1;45:7. doi: 10.1186/s13046-025-03575-5 (PMC12771992; doi:10.1186/s13046-025-03575-5)
Supplement: Supplementary file 2 — Supplementary Material 2. [file 13046_2025_3575_MOESM2_ESM.pdf]

Figure I shows the design plots representing the mean values of IVIS capture by level for each detach factor (i.e., ‘day’ and ‘cell line-treatment combination’) related to in vivo HCC xenograft orthotopic experiments B56 (left) and B76 (right). In x-axis are shown the factors (i.e., “Day” and “Cell line and treatment”), and in y-axis the IVIS capture level. The horizontal notches represent the mean values of the IVIS capture.

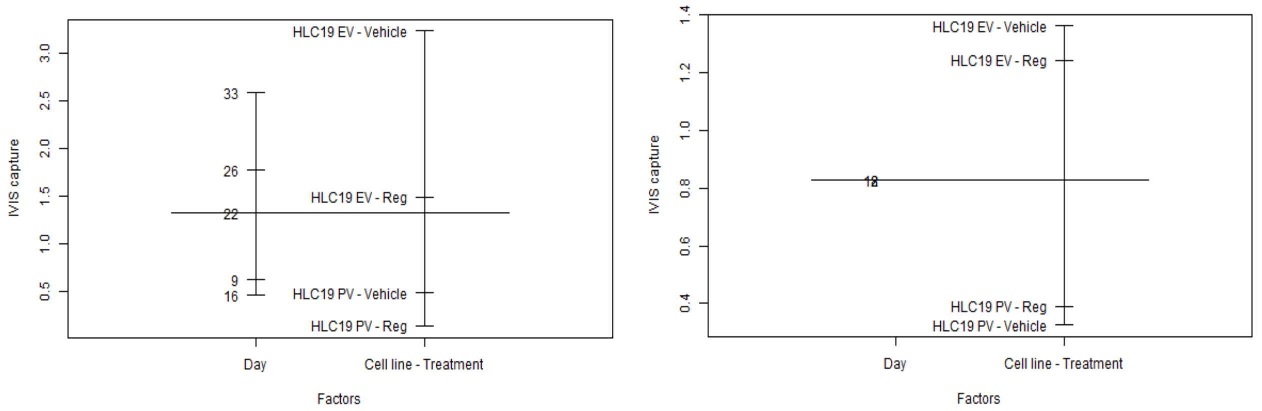

Figure I. Design plots for data related to in vivo HCC xenograft orthotopic experiment B56 (left) and B76 (right), in the right side on data X-axis: factors (i.e., “Day” and “Cell line and treatment”); Y-axis: IVIS capture level. The horizontal notches represent the mean level of the IVIS capture for each level of the factor.

Table I reports the mean (± sd) values across days and significance for each ‘cell line-treatment combination’ for B56 and B76 data. Wilcoxon test was performed by considering as time points the baseline and the last follow up values. In this way, four statistical tests were carried out: regarding B56 data, IVIS capture changes were significant only for HLC19 PV – Reg combination (P= 0.002) and suggestive within HLC19 EV – Vehicle group (P = 0.082). No significance was detected for HLC19 EV – Reg (P = 0.578) and HLC19 PV – Vehicle (P = 1). On B76, the PV groups provided significant results (P = 0.002).

**B56 data**				**B76 data**		
**Cell line and treatment**	**Day**	**IVIS capture**		**Cell line and treatment**	**Day**	**IVIS capture**
**HLC19 EV Vehicle**	**9**	0.565 ± 0.419		**HLC19 EV Vehicle**	**8**	1.147 ± 0.557
**HLC19 PV Vehicle**	**9**	0.483 ± 0.358		**HLC19 PV Vehicle**	**8**	0.504 ± 0.378
**HLC19 EV Reg**	**9**	0.8013 ± 0.556		**HLC19 EV Reg**	**8**	1.075 ± 0.717
**HLC19 PV Reg**	**9**	0.604 ± 0.377		**HLC19 PV Reg**	**8**	0.583 ± 0.626
**HLC19 EV Vehicle**	**16**	0.739 ± 1.366		**HLC19 EV Vehicle**	**12**	1.572 ± 2.779
**HLC19 PV Vehicle**	**16**	0.066 ± 0.077		**HLC19 PV Vehicle**	**12**	0.147 ± 0.319
**HLC19 EV Reg**	**16**	1.019 ± 1.747		**HLC19 EV Reg**	**12**	1.405 ± 1.764
**HLC19 PV Reg**	**16**	0.013 ± 0.029		**HLC19 PV Reg**	**12**	0.193 ± 0.346
**HLC19 EV Vehicle**	**22**	2.779 ± 3.371				
**HLC19 PV Vehicle**	**22**	0.171 ± 0.278				
**HLC19 EV Reg**	**22**	2.308 ± 3.099				
**HLC19 PV Reg**	**22**	0.004 ± 0.008				
**HLC19 EV Vehicle**	**26**	5.182 ± 6.265				
**HLC19 PV Vehicle**	**26**	0.520 ± 1.060				
**HLC19 EV Reg**	**26**	NA±NA				

**HLC19 PV Reg**	**26**	0.015 ± 0.043				
**HLC19 EV Vehicle**	**33**	6.919 ± 7.989				
**HLC19 PV Vehicle**	**33**	1.165 ± 2.491				
**HLC19 EV Reg**	**33**	NA ± NA				
**HLC19 PV Reg**	**33**	0.002 ± 0.001				

Note. The values are expressed as mean ± sd

Table I. Descriptive statistics of IVIS capture across Day and cell line treatment for B56 and B76 data.

Subsequentially, an evaluation of the synergism (vs additivity) was performed by an interaction analysis. Concerning that, Figure II, *upper*, shows the interaction plots across days of the IVIS capture values in relation to the ‘cell line-treatment combination’ on B56 data. Figure II, *lower*, shows the strong parallelism within the two cell lines (EV and PV), in decreasing way in PV and increasing in EV.

It is worth noting, the x-axis values were presented from the first time point after baseline because of lack of variability at baseline.

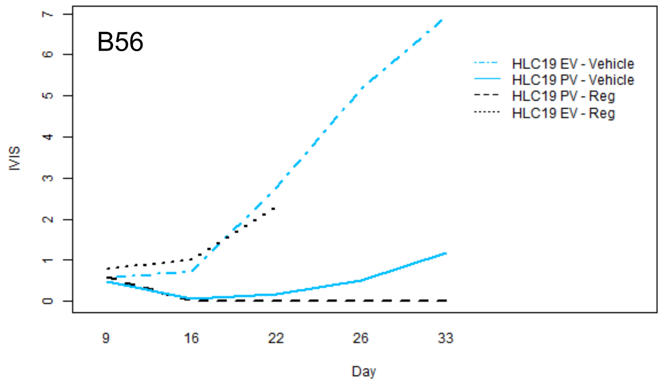

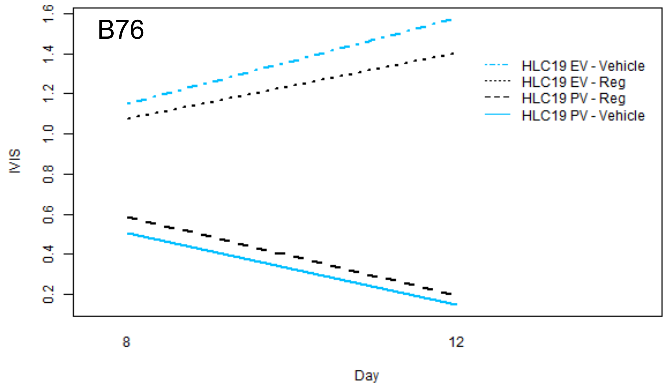

Figure II. Interaction plots for B56 an B76 data. Design plot for data of B56 (upper panel), and B76 (lower panel) experiments. X-axis: days; Y-axis: IVIS captured signal.

Regarding the GEE modelling, the tables II-VI show the results in terms of coefficients, P-value and 95% CI. Notably, table II reports the B56 results (experiment 1): firstly, the EV Vehicle level (reference category) reports an increasing trend of IVIS capture across days. Hence, the interaction (i.e., synergism) between day factor and cell line-treatment combination (ref.: EV Vehicle) was significant for PV REG (-0.311, P = 0.002) and PV Vehicle (-0.259, P = 0.016), by reporting negative values in terms of expected variation. Similarly, also the interaction with EV REG shows a negative value but in suggestive way (-0.209, P = 0.057): this result follows the interaction plot where the IVIS capture trend is less increasing than EV Vehicle modality.

Treatment – Cell line combination*	Estimate<br>P-value<br>95% CI
**Experiment 1 (outcome: B56 normalized IVIS captures, Ref: EV Vehicle)**	
**EV Reg**	**2.932**<br>**0.009**<br>**0.722; 5.144**
**PV Reg**	**3.483**<br>**0.001**<br>**1.387; 5.579**
**PV Vehicle**	**2.743**<br>**0.016**<br>**0.493; 4.992**
**Day**	**0.289**<br>**0.005**<br>**0.086; 0.493**
**EV Reg x Day (interaction)**	*-0.209*<br>*0.057*<br>*-0.425; 0.007*
**PV Reg x Day (interaction)**	**-0.311**<br>**0.002**<br>**-0.515; -0.107**
**PV Vehicle x Day (interaction)**	**-0.259**<br>**0.016**<br>**-0.471; -0.047**

Note. 95% CI: 95% confidence interval. The significant results (P < 0.05) are in **bold**, in *italic* the suggestive ones (0.01 < P < 0.05).

Table II. Results of the GEE modelling on B56 data

Thus, table III show the results of the interaction contrasts on cell line-treatment factor achieved by a multiple comparison analysis (adjusted by Bejamini-Hochberg-Yekutieli False Discovery Rate, FDR). Because the interaction contrasts are *per se* stratified, we have set as stratification day the mean day (day 20.97~21). Of note, comparisons between PV REG (3.038, P = 0.066) and PV Vehicle (2.696, P = 0.095) with EV Vehicle returned suggestive results. Similarly, EV REG vs PV REG comparison provided a relevant result (1.587, P = 0.067).

Interaction contrast (day=21*)	Estimate<br>P-value<br>95% CI
**EV - Vehicle - EV – Reg**	1.451<br>0.626

	-1.942; 4.845
**EV - Vehicle - PV – Reg**	*3.038*<br>*0.066*<br>*0.0459; 6.029*
**EV - Vehicle - PV – Vehicle**	*2.696*<br>*0.095*<br>*-0.354; 5.746*
**EV - Reg - PV - Reg**	*1.587*<br>*0.067*<br>*-0.018; 3.191*
**EV - Reg - PV – Vehicle**	1.245<br>0.198<br>-0.466; 2.955
**PV - Reg - PV - Vehicle**	-0.342<br>0.381<br>-0.941; 0.257

Note. 95% CI: 95% Confidence Interval. The significant results (P < 0.05) are in **bold**, in *italic* the suggestive ones (0.05<P<0.10). * Mean day within the follow up. The P-values are adjusted by using a Benjamini-Hochberg-Yekutieli False Discovery Rate (FDR) procedure.

Table III. Results of the multiple comparison analysis on cell line-treatment combinations (B56 data)

Figure III graphically reports the point and interval estimations (as mean difference, at 95% level) of the contrasts.

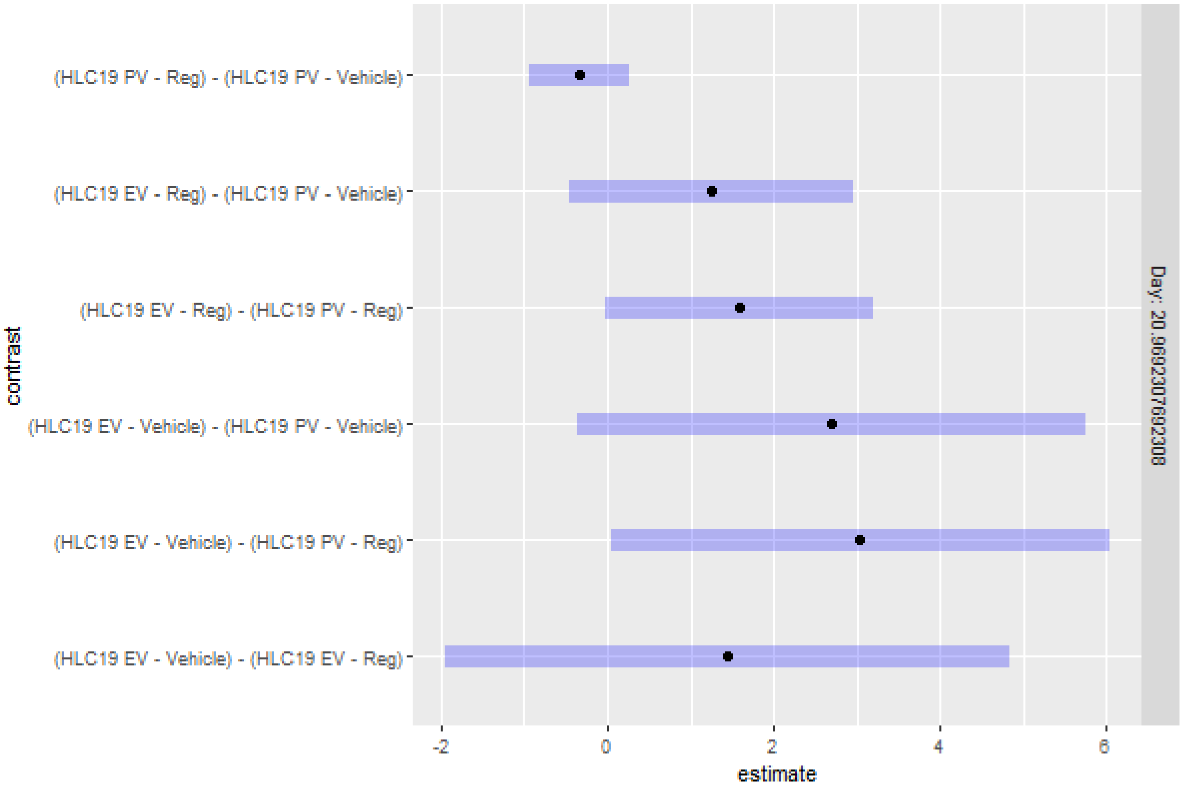

Figure III. Point and interval estimation of the multiple comparison analysis on cell line-treatment combination for B56 data.

Analogously, tables IV, V, and VI report B76 results (experiment 2): firstly, no significance was got, from both main effects (Day and cell line-treatment) and interaction term in relation to the EV Vehicle reference category

(see table IV). However, we have moved forward by performing two multiple comparison analysis (adjusted by Bejamini-Hochberg-Yekutieli FDR): i) on the cell line-treatment levels, stratified by day and *vice versa* (see table V); ii) on the days (considered as dichotomous factor), stratified by cell line-treatment levels (table VI). In the first one, just a significance was returned, on the EV Vehicle vs PV Vehicle contrast at day 8 (0.644, P = 0.032). In the second analysis, PV stratified comparisons (i.e., Day 8 – Day 12) provided significant results: within PV REG (0.390, P = 0.001) and PV Vehicle (0.356, P < 0.001).

**Experiment 2 (outcome: B76 normalized IVIS captures, Ref: EV + Vehicle)**	**Estimate<br>P-value<br>95% CI**
**EV – Reg**	0.117<br>0.94<br>-3.133; 3.367
**PV - Reg**	1.065<br>0.44<br>-1.655; 3.785
**PV - Vehicle**	0.919<br>0.49<br>-1.721; 3.559
**Day**	0.106<br>0.55<br>-0.244; 0.457
**EV – Reg x Day (interaction)**	-0.023<br>0.91<br>-0.447; 0.400
**PV – Reg x Day (interaction)**	-0.204<br>0.26<br>-0.559; 0.151
**PV – Vehicle x Day (interaction)**	-0.195<br>0.28<br>-0.548; 0.157

Table IV. Results of the GEE modelling on B76 data

Interaction contrast	Estimate<br>P-value<br>95% CI
Day 8	
**EV Vehicle vs EV Reg**	0.073<br>1.000<br>-0.667; 0.81
**EV Vehicle vs PV Reg**	0.565<br>0.136<br>-0.118; 1.25
**EV Vehicle vs PV Vehicle**	**0.644**<br>**0.032**<br>**-0.354; 5.746**
**EV Reg vs PV Reg**	0.492<br>0.329<br>-0.018; 3.191
**EV Reg vs PV Vehicle**	0.571<br>0.136<br>-0.466; 2.955
**PV Reg vs PV Vehicle**	0.079<br>1.000<br>-0.941; 0.257
Day 12	
**EV Vehicle vs EV Reg**	0.168<br>1.000<br>-2.512; 2.85
**EV Vehicle vs PV Reg**	1.379<br>0.386<br>-0.900; 3.66
**EV Vehicle vs PV Vehicle**	1.425<br>0.386<br>-0.852; 3.70
**EV Reg vs PV Reg**	1.212<br>0.204<br>-0.252; 2.67
**EV Reg vs PV Vehicle**	1.257<br>0.204<br>-0.202; 2.72
**PV Reg vs PV Vehicle**	0.046<br>1.000<br>-0.338; 0.43

Note. 95% CI: 95% confidence interval. The significant results ($P < 0.05$) are in **bold**, in *italic* the suggestive ones ($0.05 < P < 0.10$). The P-values are adjusted by using a Benjamini-Hochberg-Yekutieli FDR (False Discovery Rate) procedure.

Table V. Results of the multiple comparison analysis on cell line-treatment combinations stratified by day (B76 data)

Interaction contrast (Strata)	Estimate<br>P-value<br>95% CI
**Day8 - Day12 (EV Vehicle)**	-0.425<br>0.555<br>-1.852; 1.002
**Day8 - Day12 (EV Reg)**	-0.330<br>0.497<br>-1.294; 0.634
**Day8 - Day12 (PV Reg)**	**0.390**<br>**0.001**<br>**0.169; 0.610**
**Day8 - Day12 (PV Vehicle)**	**0.356**<br>**< 0.001**<br>**0.223; 0.489**

Note. 95% CI: 95% confidence interval. The significant results ($P < 0.05$) are in **bold**, in *italic* the suggestive ones ($0.05 < P < 0.10$). The P-values are adjusted by using a Benjamini-Hochberg-Yekutieli False Discovery Rate (FDR) procedure.

Table VI. Multiple comparison analysis on the days by cell line-treatment strata.

Figure III graphically reports the point and interval estimations (as mean difference, at 95% level) of the contrasts. Of note, it is worth to point out that the B76 data were trimmed at 12th day for experimental purposes.

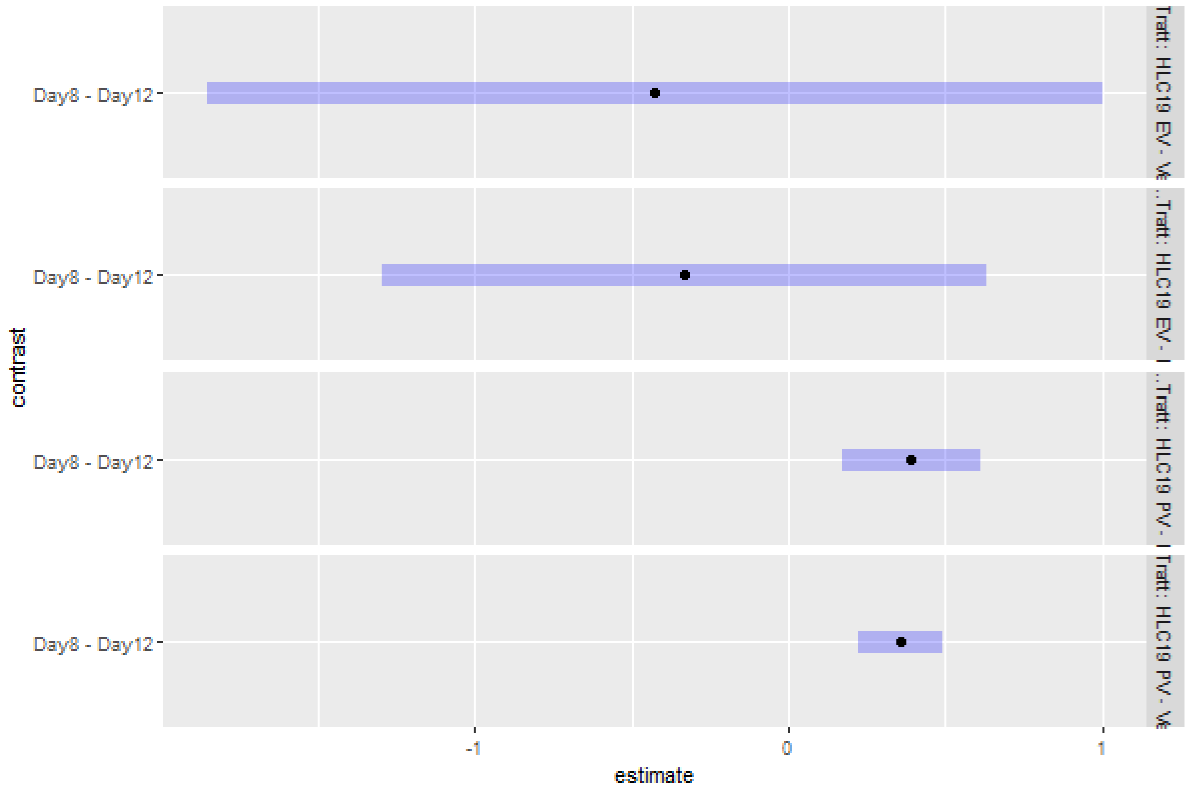

Figure III. Point and interval estimation of the multiple comparison analysis on days stratified by cell line-treatment combination (B76 data)
